# The inflammatory and genetic mechanisms underlying the cumulative effect of co-occurring pain conditions on depression

**DOI:** 10.1126/sciadv.adt1083

**Published:** 2025-04-02

**Authors:** Rongtao Jiang, Paul Geha, Matthew Rosenblatt, Yunhe Wang, Zening Fu, Maya Foster, Wei Dai, Vince D. Calhoun, Jing Sui, Marisa N. Spann, Dustin Scheinost

**Affiliations:** ^1^ State Key Laboratory of Cognitive Neuroscience and Learning, Beijing Normal University, Beijing, China.; ^2^ Department of Radiology and Biomedical Imaging, Yale School of Medicine, New Haven, CT 06510, USA.; ^3^ Department of Psychiatry, University of Rochester Medical Center, Rochester, NY 14642, USA.; ^4^ Department of Biomedical Engineering, Yale University, New Haven, CT 06520, USA.; ^5^ Nuffield Department of Population Health, University of Oxford, Oxford, UK.; ^6^ Tri-institutional Center for Translational Research in Neuroimaging and Data Science (TReNDS), Georgia State University, Georgia Institute of Technology, and Emory University, Atlanta, GA 30303, USA.; ^7^ Interdepartmental Neuroscience Program, Yale University, New Haven, CT 06520, USA.; ^8^ Department of Biostatistics, Yale University, New Haven, CT 06520, USA.; ^9^ Department of Psychiatry, Columbia University Irving Medical Center, New York, NY 10032, USA.; ^10^ Child Study Center, Yale School of Medicine, New Haven, CT 06510, USA.

## Abstract

Chronic pain conditions frequently coexist and share common genetic vulnerabilities. Despite evidence showing associations between pain and depression, the additive effect of co-occurring pain conditions on depression risk and the underlying mechanisms remain unclear. Leveraging data from 431,038 UK Biobank participants with 14-year follow-up, we found a significantly increased risk of depression incidence in individuals reporting pain, irrespective of body site or duration (acute or chronic), compared with pain-free individuals. The depression risk increased with the number of co-occurring pain sites. Mendelian randomization supported potential causal inference. We constructed a composite pain score by combining individual effects of acute or chronic pain conditions across eight body sites in a weighted manner. We found that depression risks increased monotonically in parallel with composite pain scores. Moreover, some inflammatory markers, including C-reactive protein, partially mediated the association between composite pain scores and depression risk. Considering the high prevalence of comorbid depression and pain, pain screening may help identify high-risk individuals for depression.

## INTRODUCTION

Chronic pain—defined as pain persisting for over 3 months—is a complex and heterogeneous condition (*1*) influenced by a combination of biopsychosocial, environmental, and genetic factors (*2*–*4*). Globally, about 30% of people suffer from chronic pain, making it the most frequent reason for seeking health care (*5*, *6*). Chronic pain is now recognized as a separate disease entity rather than an accompanying symptom of an underlying disease (*7*).

In addition to physical suffering, emerging evidence suggests that pain may lead to depression (*8*). Notably, chronic pain across distinct body sites (e.g., head and shoulder) has been associated with severe depressive symptoms (*8*–*11*). A meta-analysis involving data from 47 countries found that self-reported pain was related to a 3.93 times higher risk of depression (*12*). This relationship may be partly explained by the small samples examined in prior studies, which were mostly cross-sectional, the study of a single pain condition at a single body site, and the study of chronic pain compared to acute pain (pain persisting for shorter than 3 months) (*13*). Hence, the reported associations between pain conditions and depression may have been affected by unmeasured confounders or reverse causation, necessitating the correct identification of causal relationships. Further studies that comprehensively characterize pain will support the identification of high-priority pain sites for intervention.

Pain conditions do not occur in isolation but frequently coexist and share common genetic and neurobiological vulnerabilities (*8*, *14*). Over one-third of pain patients report experiencing co-occurring pain conditions worldwide. Extant studies usually investigate one single pain site at a time. Failure to account for the co-occurring nature of pain locations may lead to inflated effect sizes (*14*), as pain sites showing statistically significant associations with depression when studied alone may not prove robust when considered alongside other body sites (*15*). The National Institutes of Health Pain Consortium recognizes the concept of co-occurring pain conditions as an area of priority for additional research (*16*). Epidemiological evidence has highlighted the extra burden of co-occurring pain conditions on multiple health outcomes including cardiovascular diseases (*13*), dementia (*17*, *18*), and accelerated brain aging (*18*), which are major contributors to the onset of depression. Nevertheless, whether individuals with co-occurring pain conditions are at an increased risk of depression, compared with both pain-free and single-site pain individuals, remains unclear. Now, the number of co-occurring pain sites is used to characterize the overlapping nature of pain conditions (*3*). However, pain sites may disproportionately affect depression, given evidence that different brain structures underlie pain at distinct body sites (*19*). Hence, a composite-weighted score combining extent and duration of pain conditions may show increased accuracy in capturing the additive effect of co-occurring site-specific pain conditions on depression (*20*, *21*).

Despite the extensive literature on the association between pain and depression, limited research examining the underlying mechanisms exists. Potential mechanisms include shared risk factors (including low educational attainment, obesity, physical inactivity, and social isolation) (*22*), genetic overlap [pain conditions and depression have a high genetic correlation (*8*), and common genetic and epigenetic modifications can mediate the pain-depression interaction (*23*)], the dysfunction of the serotonergic system (*24*), and disrupted neural circuits (pain and depression share overlapping brain regions, and alterations in brain regions responsible for pain processing may contribute to aberrant emotional regulation in depression) (*24*). Moreover, both chronic pain and depression have been linked to increased levels of inflammatory markers (*25*). Chronic pain is accompanied by the release of proinflammatory cytokines and activation of innate immune cells (*26*, *27*), and inflammation has an important role in the induction and maintenance of chronic pain (*28*). Similarly, inflammatory dysregulation has also been implicated in the pathophysiology of depression (*29*–*31*). This suggests that inflammation markers may be one mechanism mediating the risk of increased depression in individuals suffering from chronic pain. Elucidating this mechanism can in turn provide a better understanding of the contributing factors to depression in patients with chronic pain.

Leveraging data from UK Biobank, we systematically investigated the prospective associations between pain conditions and depression risk and examined causal inferences using Mendelian randomization (MR) (*32*). MR uses genetic variants as instrumental variables and is less likely to be affected by residual confounding effects and reverse causality. By combining distinct pain sites and duration into a composite score, we examined the additive effect of co-occurring pain conditions on depression incidence. Last, we explored inflammation markers as a potential mechanism driving the association between pain and depression.

## RESULTS

### Study sample

**Fig. 1.**
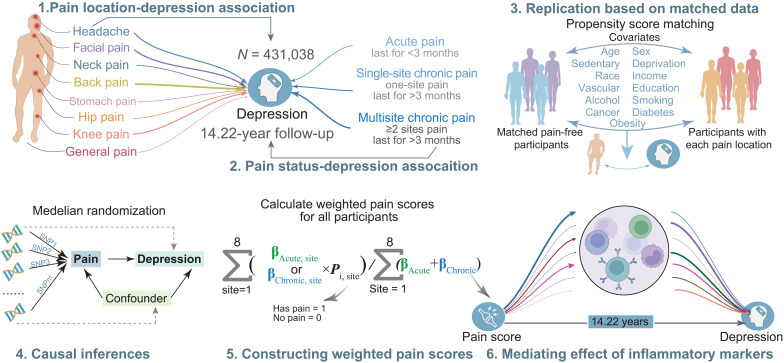
Overview of study workflow. Leveraging 431,038 participants from UK Biobank with a 14.22-year follow-up, this study investigated the prospective association between pain conditions and depression risks, which were further replicated on matched data using a propensity score matching procedure. We also used MR to make causal inferences about the effect of pain conditions on depression. We then examined the independent and joint associations of pain conditions across eight body sites and varying duration with depression incidence. Last, using 14 inflammatory markers extracted from hematological assays, we explored inflammatory markers as a potential mechanism driving the association between pain and depression.

A total of 431,038 UK Biobank depression-free participants at baseline assessment were included in this prospective study: 177,865 were pain-free, 70,964 reported acute pain, and 182,209 reported chronic pain. Of the participants reporting specific pain site(s) (excluding participants with general pain), 99,383 reported single-site chronic pain (SCP), and 76,756 reported multisite chronic pain (MCP). Baseline characteristics by pain status are shown in tables S1 and S2. Overall, individuals had a mean age of 56.6 years at baseline and 53.21% were female. Compared with pain-free individuals, those with pain were more likely to be females, materially deprived, of a non-white race, current smokers; they also drank less frequently, had lower household income, had lower educational attainment, reported more sedentary behavior, and had a higher prevalence of vascular problems, obesity, and diabetes. Figure 1 shows an overview of analyses performed in the current study.

### Associations of pain across eight body sites with depression risks

**Fig. 2.**
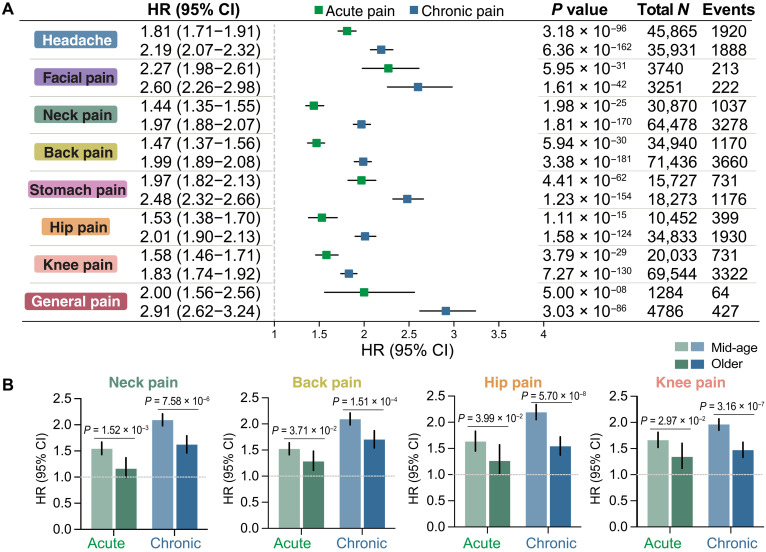
Prospective associations of acute or chronic pain across eight body sites and incidence of depression. (**A**) Compared with pain-free individuals, those with pain showed a significantly increased risk of developing depression, irrespective of body site, duration, and adjustment for multiple covariates. (**B**) Interaction analyses supported a significantly modifying effect of age on the association of pain in the neck, back, hip, and knee with the depression risk, with participants aged younger than 65 years showing a higher depression risk than their older counterparts. The error bar and horizontal lines indicate the corresponding 95% CI. The size of the bars and the internal center represent mean HRs.

During a median follow-up of 14.22 years [interquartile range (IQR) 13.50 to 14.91 years], 13,947 individuals developed depression. Compared with pain-free individuals, those with pain showed a significantly increased risk of developing depression after adjustment for important covariates (e.g., age; sex; race; material deprivation; educational attainment; household income; smoking status; alcohol intake frequency; sedentary behavior; BMI category; and history of cancer, vascular or heart problems, and diabetes), irrespective of body site or duration (acute or chronic; Fig. 2A). For each pain site, the strength of association was larger in chronic pain than in acute pain. Notably, participants reporting chronic general pain showed the highest risk of depression incidence {hazard ratio (HR) = 2.91, 95% confidence interval (CI) = [2.62, 3.24], *P* = 3.03 × 10^−86^}, followed by chronic facial pain (HR = 2.60, [2.26, 2.98], *P* = 1.61 × 10^−42^), chronic stomach pain (HR = 2.48, [2.32, 2.66], *P* = 1.23 × 10^−154^), and headache (HR = 2.19, [2.07, 2.32], *P* = 6.36 × 10^−162^). All associations survived correction for multiple comparisons (Bonferroni-corrected significance threshold *P* < 0.05/8), persisted in subgroups stratified by covariates (fig. S1) and attenuated slightly when using a 10-year landmark analysis (fig. S2) or when adding pain medication as a confounder (e.g., aspirin, ibuprofen, and paracetamol; fig. S3). Moreover, excluding participants having anxiety at baseline from the analyses did not appreciably change the associations (fig. S4).

Interaction analyses support a significant modulating effect of age on the association of pain in the neck, back, hip, and knee with depression risks (Bonferroni-corrected *P* threshold < 0.05/13 for 13 tests), but not for the other covariates (fig. S5). In stratified analyses, individuals with acute or chronic pain at these four sites aged younger than 65 years had a higher depression risk than those aged 65 years and older (Fig. 2B).

### Associations between pain status and depression risks

**Fig. 3.**
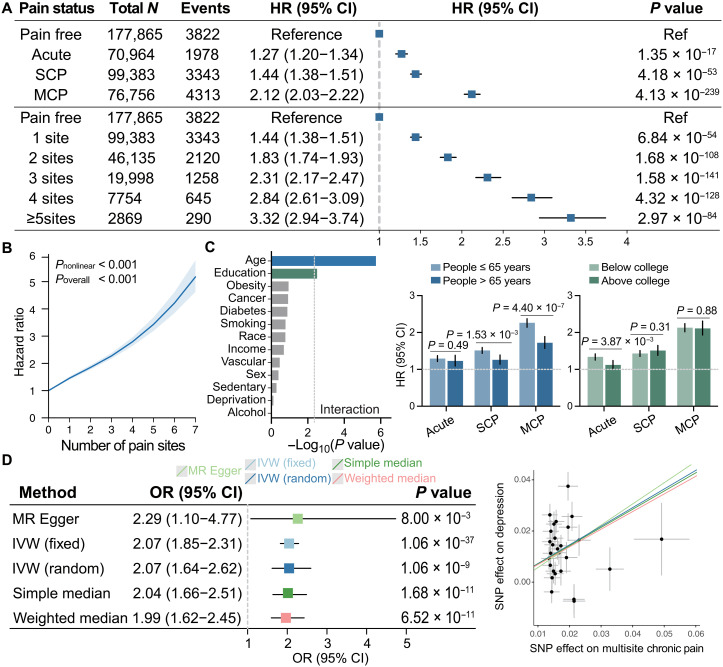
Prospective and causal associations of MCP with incident depression. (**A**) Compared with pain-free individuals, those with acute pain, SCP, and MCP had a higher depression risk after adjustment for covariates and multiple comparisons, and depression risks increased along with the number of co-occurring pain sites. (**B**) Exposure-response curve between the number of co-occurring pain sites and depression risks was nonlinear, with plateauing trends at lower exposure but steeper slopes at higher exposure. (**C**) Significant interaction between pain status and age and between pain status and education attainment was observed. The dashed vertical line indicates the significance threshold after correcting for multiple testing [−log(0.05/13) =2.41]. Specifically, the associations tended to be higher in people younger than 65 years for SCP and MCP. Individuals with acute pain having a college degree showed a higher risk of depression than those without a college degree. (**D**) MR estimates provided evidence for a significantly detrimental effect of MCP on depression. The scatterplot shows SNP effects on MCP and depression.

Compared with pain-free individuals, those with pain showed a significantly increased risk of depression incidence after adjustment for covariates and multiple comparisons, which was 1.27 times higher in acute pain (95% CI = [1.20, 1.34], *P* = 1.35 × 10^−17^), 1.44 times higher in SCP (95% CI = [1.38, 1.51], *P* = 4.18 × 10^−53^), and 2.21 times higher in MCP (95% CI = [2.03, 2.33], *P* = 4.13 × 10^−239^) (Fig. 3A and table S3). A dose-response relationship was observed for this association with participants experiencing one to five or more co-occurring pain sites having a 1.44-, 1.83-, 2.31-, 2.84-, and 3.32-fold increased depression risk compared with pain-free individuals. The exposure-response curve between the number of co-occurring pain sites and depression risks was nonlinear (*P* < 0.001; Fig. 3B), with plateauing trends at lower exposure but steeper slopes at higher exposure. Notably, MCP demonstrated a 47% higher depression risk than SCP (HR = 1.47, 95% CI = [1.41, 1.54], *P* = 9.58 × 10^−63^). Associations persisted in subgroups stratified by covariates (fig. S6). Sensitivity analyses, including a 10-year landmark analysis, excluding participants having anxiety at baseline, and additional adjustments for pain medications, did not appreciably change the associations (figs. S2 to S4).

Significant pain status-age and pain status-educational attainment interactions were observed (Fig. 3C). These associations tended to be higher in people younger than 65 years as compared to their older counterparts for SCP (*P* = 1.53 × 10^−3^) and MCP (*P* = 4.40 × 10^−7^). In contrast, the modifying effect of education was only significant for participants experiencing acute pain, with individuals having a college degree showing a higher depression risk than those without a college degree (*P* = 3.87 × 10^−3^; table S4).

### Replication of associations in matched data

For each of the eight acute or chronic pain sites, the propensity score matching (*33*) selected an equal number of controls from the pain-free individuals, matched on all covariates with the corresponding pain group (all *P* > 0.05; fig. S7 and table S5). When the Cox proportional analyses were restricted to the matched data, similar trends of associations regarding the direction and magnitude of associations were observed between acute or chronic pain conditions across all sites and depression incidence (fig. S8). On the basis of matched data, the risk of depression in participants with acute pain, SCP, and MCP was also similar with that from the main analyses, validating the robustness of the results.

### Causal inferences of the effect of pain on depression

The MR estimates provided evidence for a detrimental effect of MCP on depression but not for pain at any specific body site (table S6). Specifically, using MCP-associated single-nucleotide polymorphisms (SNPs) as proxies (table S7), the inverse-variance weighted (IVW) method under random effect found a 2.07 times higher risk of depression per one-point increment in co-occurring pain sites [odds ratio (OR) = 2.07, 95% CI = [1.64, 2.62], *P* = 1.06 × 10^−9^, Fig. 3D)]. The estimation based on other MR methods including weighted median (OR = 1.99, 95% CI = [1.62, 2.45], *P* = 6.52 × 10^−11^) simple median (OR = 2.04, 95% CI = [1.66, 2.51], *P* = 1.68 × 10^−11^), MR Egger (OR = 2.29, 95% CI = [1.10, 4.77], *P* = 8.00 × 10^−3^), and IVW under fixed effect (OR = 2.07, 95% CI = [1.85, 2.31], *P* = 1.06 × 10^−37^) revealed comparable effect sizes, but the MR Egger method was only marginally significant (Bonferroni-corrected significance threshold *P* < 0.05/9). The scatterplot of SNP effects on MCP and depression is shown in Fig. 3D.

The MR Egger intercept test suggested no obvious directional pleiotropy (intercept = 0.012, *P* = 0.056), but Cochran’s Q test indicated significant heterogeneity (*Q* = 106.02, *P* = 5.95 × 10^−12^). Therefore, the IVW method under random effect was used as the primary method. Moreover, MR pleiotropy residual sum and outlier (MR-PRESSO) detected four outlying variants and removing these outliers nominally impacted the estimation (OR = 2.06, 95% CI = [1.70, 2.50], *P* = 2.20 × 10^−13^). Analyses leaving out each SNP revealed that no single SNP drove the estimation (fig. S9). These results were consistent with those using summary statistics for depression from a different consortium (fig. S10 and table S8).

### The joint effect of pain conditions on incident depression

**Fig. 4.**
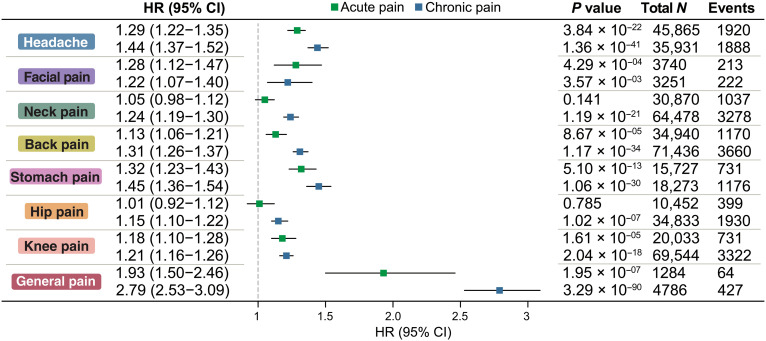
The independent association between each pain condition and the risk of depression after mutually adjusting for the other pain conditions. Dots indicate mean HRs, and the horizontal lines indicate the corresponding 95% CI.

Mutual adjustment by all pain sites in a single model revealed significantly attenuated associations between site-specific pain conditions and depression risk compared with unadjusted models (Fig. 4). Individuals with chronic pain across all regional body sites had a significantly higher risk of depression in comparison to pain-free individuals, with HRs ranging from 1.15 (hip pain) to 1.45 (stomach pain). Individuals with acute pain also showed an increased depression risk than pain-free individuals, although the effect size for each acute pain site was lower than its corresponding chronic pain site. The risk of depression for acute pain in the neck (*P* = 0.141) and hip (*P* = 0.785) fell short of significance. Of the seven localized body sites, participants reporting stomach pain (HR = 1.45, [1.36, 1.54], *P* = 1.06 × 10^−30^) and headaches (HR = 1.44, [1.37, 1.52], *P* = 1.36 × 10^−41^) had comparably highest risks. Pain at the other body sites showed similar effect sizes.

**Fig. 5.**
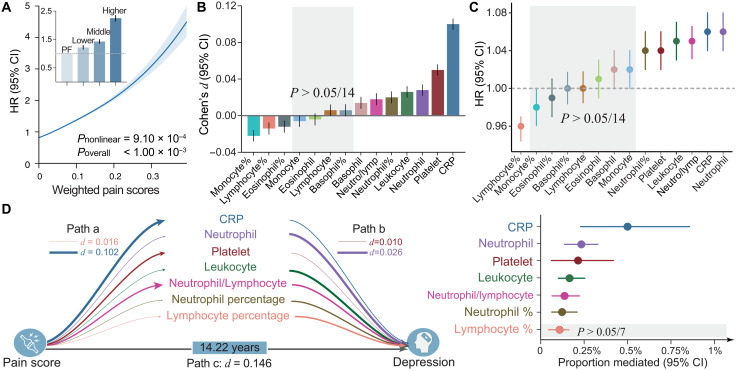
Association of inflammatory markers with composite pain scores and depression incidence. (**A**) Strong evidence supported a nonlinear association between composite pain scores and depression risks, with linear trends at lower exposure and steeper slopes at higher exposure. Compared with pain-free individuals, those in the lowest, middle, and highest tertile of composite pain scores demonstrated a 1.22-fold, 1.43-fold, and 2.25-fold elevated risk of depression incidence, respectively. (**B**) Of all 14 inflammatory markers, 10 showed significant associations with composite pain scores after controlling for covariates and multiple comparisons. (**C**) Significant linear associations were also observed between 7 of the 14 inflammatory markers and incident depression after controlling for numerous covariates and multiple comparisons. The size of the bars and the internal center represent mean HRs. (**D**) Mediation analyses showed that six of these seven inflammatory markers significantly and partially mediated the prospective association between composite pain scores and depression incidence while controlling for numerous covariates and multiple comparisons. Dots indicates mean HRs; vertical and horizontal lines indicate 95% CI. Path thickness indicates the strength of associations, and numerical values for the largest and smallest effect sizes are provided for reference.

The composite pain scores were calculated by combining the individual effects of acute or chronic pain across all body sites (fig. S11). Strong evidence supported a nonlinear positive association between composite pain scores and depression risks (*P* = 9.10 × 10^−4^). The risk of depression increased monotonically across the entire range of composite pain scores with a stronger detrimental effect at higher exposure (Fig. 5A). Similar patterns of associations were observed when investigating the tertile of the composite scores. Specifically, compared with pain-free individuals, those in the lowest, middle, or highest tertile of composite pain scores demonstrated a 1.22- (95% CI = [1.16, 1.29], *P* = 7.68 × 10^−14^), 1.43- ([1.36, 1.50], *P* = 8.86 × 10^−47^), and 2.25-fold ([2.15, 2.35], *P* = 1.96 × 10^−293^) elevated risk of depression, respectively (table S9). The three groups of covariates (socioeconomic, lifestyle, and physical health) showed comparable effect sizes in explaining the associations of the composite pain scores and depression incidence (fig. S12).

### The mediating effect of inflammatory markers

Of all 14 inflammatory markers, 10 showed Bonferroni-corrected significant associations with composite pain scores after controlling for covariates (Fig. 5B and table S10). C-reactive protein (CRP) had the largest effect size (Cohen’s *d* = 0.10, 95% CI = [0.094, 0.106], *P* = 1.51 × 10^−258^). MCP showed stronger associations with most inflammatory markers than SCP (fig. S13). Significant linear associations were also observed between seven of the 14 inflammatory markers and incident depression after controlling for covariates and multiple comparisons (Fig. 5C and table S11). More specifically, the neutrophils, CRP, neutrophil-to-lymphocyte ratio, leukocytes, platelet, and neutrophil percentage showed detrimental effects, but the lymphocyte percentage showed protective effects. No evidence of nonlinearity was observed for any inflammatory markers.

Mediation analyses were further performed on these seven inflammatory markers showing significant associations with both composite pain scores and depression incidence. We found that six of these seven inflammatory markers (except lymphocyte percentage) significantly and partially mediated the prospective association between composite pain scores and depression incidence while controlling for covariates and multiple comparisons (bootstrapping test, *P* < 2.0 × 10^−4^; Fig. 5D), and CRP showed the greatest proportion of mediated variance.

## DISCUSSION

In this study, individuals in the UK Biobank with either acute or chronic pain across all body sites were at a higher risk of developing depression during a 14-year follow-up period. We also found an additional burden and causal inference of co-occurring pain conditions on depression risk. Moreover, CRP, neutrophil, platelet, leukocytes, and neutrophil-to-lymphocyte ratio significantly mediated the association between pain and depression, suggesting underlying mechanisms driven by systemic inflammation.

Our findings corroborate existing results demonstrating positive associations between pain conditions and depression (*22*, *34*). Previous studies have generally focused on patients with chronic pain, leaving the association between acute pain and depression largely unknown. Acute pain may be overlooked in depression research because it is often associated with tissue damage that disappears upon tissue healing (*35*). Yet, our study indicated significantly detrimental effects of acute pain on depression risk, with HRs ranging from 1.44 to 2.27 depending on different body sites. Despite a smaller magnitude than the corresponding chronic pain condition, the increased hazards conferred by acute pain were comparable with and even greater than some well-established risk factors of depression such as sedentary behavior (*36*), physical inactivity (*37*), and sleep problems (*15*). In this regard, acute pain may provide a unique opportunity for early intervention to delay the onset of depression before its progression to a chronic stage, which is more challenging to manage (*1*).

Chronic overlapping pain conditions are known to be associated with negative affect such as depression or anxiety (*14*). Our results lend strong support to this association and demonstrate both a steep accentuation of the risk of depression with the increased number of sites and a potential underlying genetic causal effect leading from MCP to depression. Hence, MCP exhibited stronger associations with depression than SCP. This can be partly explained by their different neurobiological correlates. A recent study revealed significantly reduced hippocampal gray matter volume in MCP individuals, but not in SCP individuals (*17*). The hippocampus is implicated in multiple depression-related processes (*38*), and the accelerated brain aging of hippocampus in MCP may be the underlying cause of depression. MCP has a much stronger genetic component than SCP (*39*), which may increase the vulnerability to depression. Furthermore, MCP may have distinct pathophysiology from SCP. Specifically, the top two genes specific to MCP were colorectal cancer suppressor (DCC) and sidekick cell adhesion molecule 1 (SDK1) (*39*), which were enriched in subcortical limbic regions and were involved in mechanisms related to axonogenesis. This may contribute to emotional dysregulation in depression. Moreover, our additional analyses indicated that systemic inflammation may play a more important role in MCP than in SCP, with MCP showing stronger associations with most inflammatory markers than SCP (fig. S13). Furthermore, using MR with genetic instruments selected from large-scale genome-wide association studies (GWAS), we found evidence supporting a potential causal effect for genetically predicted MCP, but not for any site-specific pain, on depression. This result highlights the importance of a comprehensive approach to patients with chronic pain accounting for all painful body sites and suggests that the development of depression may stem from the cumulative effect of multiple overlapping pain conditions (*23*). Our causal estimation was similar to that observed in two recent MR studies (*23*, *40*). Nevertheless, overfitting might be a common concern in these studies because they used overlapped samples to generate summary statistics for pain and depression.

Given the strong effects of MCP on pain, future pain research should incorporate a dimensional rather than a categorical approach. Consistent with this approach, we constructed a composite pain score by combining the individual effects of acute or chronic pain conditions across eight body sites. Using the composite score is a more comprehensive approach than simply using the number of co-occurring conditions (*41*) because the composite pain scores combined these individual pain conditions in a weighted manner factoring in their disproportionate effects on depression incidence (*42*, *43*). Depression risks increased monotonically alongside increasing composite pain scores, lending support to the hypothesis that pain exists on a “continuum of widespreadness” (*44*).

Although the prevalence of pain increases with age, middle-aged adults with chronic pain of the neck, back, hip, or knee had a greater risk of developing depression than their older counterparts. This can be partly attributed to middle-aged adults having an enhanced sensitivity to physiological pain, which may further translate into negative emotions (*45*). Moreover, older adults are more likely to benefit from more effective coping strategies in regulating unpleasant emotional experiences resulting from pain (*46*). We also observed a higher risk of depression with acute pain in people who had no college education. This observation may be attributed to disparities in access to health care. Together, these findings emphasize the need for an early assessment and intervention in middle-aged patients and/or patients with less than a college education suffering from a new bout of pain to prevent the onset of mood symptoms.

Our study also provided preliminary evidence for a mechanistic explanation for the link between pain and depression. We demonstrated that inflammatory markers, particularly CRP, significantly mediated the effect of composite pain scores on depression risks, indicating a critical role of systemic inflammation. Chronic pain is associated with inflammatory dysregulation and can stimulate the release of proinflammatory cytokines, which further lead to the activation of resident glial cells (*47*) and result in neuroinflammation (*28*, *48*, *49*). Neuroinflammation can destroy synaptic plasticity (*50*) and impair neurogenesis (*51*) by increasing the concentration of neurotoxic metabolites in the brain and limiting the transmission and transport of glutamate, which leads to the development of depression (*52*). Proinflammatory cytokines can also lead to depression-like behaviors by damaging the blood-brain barrier and causing hypothalamic-pituitary-adrenal axis activation (*30*). However, it should be noted that the mediating effect was small, consistent with other large-scale studies (*17*, *53*). A primary reason is our adequate adjustment for multiple confounds co-occurring with pain and depression, which may also be linked to depression through the same inflammatory pathways. Hence, the mediating effect should be interpreted as a unique contribution beyond what can be explained by other factors.

Our findings have potential implications for public health and clinical care. Because of the significant increase in the risk of depression in individuals with pain, early screening for pain conditions may be an effective way to identify high-risk individuals who may benefit from intensive preventive efforts. Because of the scarcity of curative treatment (*54*, *55*), exploring modifiable risk factors for primary prevention is still the most crucial way to mitigate depression risk (*20*), although those factors remain limited. In addition, unlike current practices where patients with refractory chronic pain are referred to psychological medicine late in the process after having received care from multiple pain providers, early psychological assessment becomes a key step in the long-term prevention of depression onset. However, in clinical practice, a given pain condition is usually considered a symptom of a localized somatic disease (*56*, *57*). Hence our study argues for the incorporation of pain assessment into current depression management pathways (*12*).

Our analysis has some limitations. First, the pain conditions were self-reported and broadly defined from a nonstandard pain-related questionnaire. Detailed evaluation of the exact duration and intensity of pain was unavailable in UK Biobank at the baseline assessment (*8*). Future studies should incorporate improved pain assessments into calculating composite pain scores. Second, although the largest existing GWAS of pain conditions was used, some identified few significant SNPs and did not explain a significant amount of the total variance (*41*). These SNPs may not be exact proxies of pain conditions. Thus, updated MR analyses are needed as more powered genetic findings emerge. A direct study of the causal relationship between MCP and depression requires further confirmation before it is targeted in resource-intensive trails (*15*). Third, the list of included inflammatory markers is not exhaustive, only mediated a small percentage of the association between pain and depression. Inflammatory markers that are more sensitive to pain-related immunoinflammatory processes should be studied to better characterize the mediating role of systemic inflammation in the pain-depression associations. Furthermore, the mediation effects are strictly measures of association. Consequently, the mediating role of inflammatory markers in the association between pain conditions and depression warrants further validation using biological experiments. Whether intervention strategies targeting the identified inflammatory markers could help reduce depression risk attributed to chronic pain also merits further investigation (*41*). Fourth, the influence of the co-occurring patterns of pain conditions on depression incidence was not investigated. A recent study revealed that the co-occurrence of pain sites was not random but with a strong dependence between proximal body sites (*3*). Future studies can determine which type of combination of pain sites has the greatest impact on depression risks. Fifth, data in UK Biobank are limited to people of European white ancestry aged 40 years and over. Considering evidence suggesting ethnic differences in pain pathophysiology (*58*), replication and generalization of the observed associations are essential when more diverse samples become available. Last, although we adjusted for numerous confounders, unmeasured or residual confounding is inevitable and may inflate the observed associations.

In conclusion, our study demonstrates associations between depression and acute or chronic pain across all body sites, with head and stomach pain showing the strongest associations, highlighting the differential burden of pain sites on mental health. It reveals that co-occurring multiple pain conditions is prospectively and causally associated with an increased risk of depression, which is mediated by inflammatory markers. Considering the high prevalence of comorbid depression and pain, pain screening may help identify individuals at high risk for depression.

## MATERIALS AND METHODS

### Study population

The UK Biobank project is a population-based cohort study. Details are described elsewhere (*59*). In brief, more than 500,000 participants aged 37 to 73 years attended one of 22 assessment centers across the UK from 2006 to 2010, where they completed touchscreen questionnaires, had physical measurements taken, and provided biological samples. Participants for this study were excluded if they withdrew from UK Biobank, were younger than 40 years, had a history of depression at baseline, and had missing data or responded “prefer not to answer” or “do not know” for pain questionnaires or any covariates. A flowchart illustrating the criteria for sample selection is provided in fig. S14. The UK Biobank study was approved by the North West Multicenter Research Ethics Committee (no.11/NW/0382), and written informed consent was obtained from all participants.

### Pain assessment

At baseline, participants were asked whether they experienced pain that interfered with their usual activities. Possible options included pain at any seven body sites (head, face, neck/shoulder, back, stomach/abdomen, hip, and knee), “pain all over the body” (general pain), and “none of the above.” Participants could choose more than one body site for any of the seven regional body sites, but only one selection was allowed for general pain (*60*). Participants reporting pain were further asked whether this pain had lasted for more than 3 months, which is an established threshold by the International Association for the Study of Pain to distinguish chronic pain and acute pain.

The number of co-occurring chronic pain sites was then calculated into a score ranging from 0 to 7. As per previous studies (*17*), participants who reported general pain were excluded from the calculation due to the lack of site-specific pain information. Participants reporting chronic pain at a single body site were labeled as patients with SCP, while those who reported chronic pain at two or more body sites were considered as having MCP (*40*, *61*). Participants who reported “none of the above” were categorized as pain-free controls.

### Depression ascertainment

The diagnosis and date of depression were confirmed according to the ‘first occurrence of health outcomes’ category from UK Biobank database (Category 1712), which was identified through linkage to National Health Service hospital admission, death records, primary care, and self-reports from nurse-led interviews (based on physical diagnosis) (*62*). Depression was defined as ICD-10 codes F32 (depressive episode) or F33 (recurrent depressive disorder). Participants were followed up until the date of first diagnosis of depression, death, loss to follow-up, or the censoring date, whichever came first.

### Peripheral inflammation markers

Inflammatory markers were obtained from each participant with baseline hematological assays taken at recruitment. Details about the hematological analyses can be found at https://biobank.ndph.ox.ac.uk/showcase/ukb/docs/haematology.pdf. We included a total of 14 immunoinflammatory markers including CRP; platelets count; leukocytes count; and count of basophils, eosinophils, lymphocytes, monocytes, and neutrophils and their percentages in leukocytes. We also included the neutrophil-to-lymphocyte ratio to reflect the systemic inflammation status. CRP and neutrophil-to-lymphocyte ratio were log-transformed to account for significantly skewed distributions.

### Covariates

A total of 13 covariates collected at baseline were selected on the basis of a recently published study investigating the association between pain conditions and dementia incidence in the UK Biobank (*17*). All these covariates have individually been implicated in depression and may influence the association between pain conditions and depression (*62*–*64*). Sociodemographic factors included age, sex, race (white versus ethnic minorities), educational attainment (a categorical variable indicating whether participants’ highest education attainment was below or above college degree), average household income (a categorical variable with income levels from low to high; participants reporting “unknown” were created as a separate group to maximize power), and material deprivation (measured using the Townsend deprivation score and divided into tertiles with higher values indicating fewer resources). Lifestyle factors included smoking status (never, previous, and current), alcohol intake frequency (a categorical variable ranging from “never” to “daily or almost daily”) and sedentary status [determined from television watching time and categorized using 4 hours/day as a cutoff (*65*)]. Physical health factors included weight status (calculated from body mass index and categorized as underweight, normal weight, overweight, and obese according to WHO criteria; underweight and normal weight were merged due to small sample sizes), history of vascular or heart problems (determined from a self-report question indicating whether participants had the following conditions: heart attack, angina, stroke, or hypertension), history of cancer, and history of diabetes (determined from a combination of self-reporting a diagnosis of diabetes and glucose > 5.6 mM). UK Biobank field identifications for all variables used in this study are described in table S12.

### Statistical analysis

#### Association analyses between pain conditions and depression incidence

Baseline characteristics of the study populations are presented as mean with SD for continuous variables or as frequencies with percentages for categorical variables. A series of Cox proportional hazards regression models were implemented to estimate the association of pain conditions across eight body sites and pain status (SCP and MCP) with the risk of depression incidence using the follow-up time as the underlying timescale. The analyses used pain-free individuals as the reference group and adjusted for the 13 covariates described above. These covariates showed acceptably weak multicollinearity, as indicated by variance inflation factor values < 1.5. Results were reported as HRs and their 95% CIs. The potential nonlinearity was formally examined by introducing a restricted cubic spline into the Cox models. All analyses were performed using a 5-year landmark analysis to limit possible reverse causality by excluding participants experiencing events during the first 5-year follow-up (*66*). The proportional hazard assumption was checked using Schoenfeld residuals, and no violation was found. The modifying effect of all covariates on the association was examined by including a multiplicative interaction term, and stratified analyses were further performed by covariates showing significant interaction effects.

#### Replication analyses of associations using matched data

To validate the robustness of the identified associations, we used a propensity score matching procedure to control for differences in baseline characteristics between comparison groups (*67*). For each of the 16 pain conditions (8 sites × 2 duration) and two pain statuses (SCP and MCP), each participant in the pain group was matched on all 13 covariates to a single pain-free participant. We used “matchit” in the “MatchIt” package (*68*) with a logit caliper width of 0.002 and the nearest-neighbor method (*69*). We repeated the above analyses using the matched data to investigate the associations between pain conditions and depression risks.

#### Independent and additive effects of pain conditions on depression incidence

The independent association of each of the 16 pain conditions (8 body sites × 2 durations) with the risk of depression was investigated by mutually adjusting for the other 15 pain conditions. Specifically, each of the eight pain sites was dummy coded into two binary variables, one signifying the presence of acute pain, the other one signifying the presence of chronic pain. A single Cox proportional hazards model was established by simultaneously including all 16 dummy variables and 13 covariates (*20*, *21*). Then, each of the dummy variables was multiplied by the corresponding β coefficient from the mutually adjusted model, summed, and divided by the sum of all β coefficients, generating a weighted composite pain score (*21*). Theoretically, the pain-free group will have a score of 0, and a higher composite score would represent more exposure to pain risks. Last, we implemented Cox proportional hazards models to investigate the association between the composite pain score and depression risks.

#### Causal inferences using MR

We performed two-sample MR analyses to make causal inferences about the effects of genetically predicted pain conditions on depression using the “TwoSampleMR” package in R (*70*). Summary statistics for pain at the eight body sites and MCP were obtained from large GWAS performed in the European population from UK Biobank (*N* = 151,922 to 224,073 for regional pain and *N* = 387,649 for MCP) (*8*, *60*). Detailed information and source of GWAS data are provided in table S13. For each of the nine pain conditions (eight pain sites and MCP), we extracted significant SNPs based on the genome-wide significance threshold at *P* < 5.0 × 10^−8^ and relaxed the threshold to *P* < 5.0 × 10^−7^ for traits lacking enough SNPs (<3 SNPs). The extracted SNPs were used as instrument variables and clumped for independence at *r*^2^ > 0.001 with a window of 10,000 kb. The summary statistics for depression were obtained from the iPSYCH consortia (*71*) that left out samples from UK Biobank and 23andMe, which included 166,773 cases and 507,679 controls. We then harmonized the exposure and outcome datasets and used the IVW method under fixed or random effects as the primary analysis to combine effects of each SNP. Weighted median, simple median, and MR-Egger were implemented as sensitivity approaches.

We checked for evidence of heterogeneity and horizontal pleiotropy using Cochran’s *Q* test and MR-Egger regression intercept term. We also applied MR-PRESSO to detect and correct for any outliers. Leave-one-SNP-out analysis was performed to assess if the overall effect was driven by any single SNP (*15*). For sensitivity analyses, we reran the MR analyses using summary statistics for depression from the Psychiatric Genomics Consortium of individuals of European descent, which did not include UK Biobank or 23andMe data (*72*). We adhered to the guidance of the Strengthening the Reporting of Observational studies in Epidemiology (STROBE)-MR guidelines in reporting the MR study (table S14).

#### Mediating analyses of the effects of inflammatory markers

We investigated how the composite pain scores relate individually to the 14 inflammatory markers through separate linear-mixed effect models. For each model, the composite pain scores and covariates were fitted as independent fixed effects, the UK Biobank assessment center was used as a random effect, and the inflammatory markers were used as the dependent variable. The standardized β coefficients were extracted and converted to Cohen’s *d* according to previous studies (*73*, *74*). Linear and nonlinear associations between these inflammatory markers and depression incidence were also investigated using Cox proportional hazards models within the same analytical framework.

We further used the “mediation” package in R to examine whether the prospective association between composite pain scores and depression risks was mediated by inflammatory markers. We established a standard three-variable path model, where linear regression was used for pain-inflammatory associations and survival regression was used for pain-depression and inflammation-depression associations (*75*). The significance of mediating effects was determined on the basis of 5000 bootstrap iterations. All analyses were conducted in R 4.3.3, were adjusted for the same set of covariates, and were corrected for multiple comparisons using Bonferroni correction.

## References

[1] S. P. Cohen, L. Vase, W. M. Hooten, Chronic pain: an update on burden, best practices, and new advances. Lancet 397, 2082–2097 (2021). 34062143 10.1016/S0140-6736(21)00393-7

[2] S. Toikumo, R. Vickers-Smith, Z. Jinwala, H. Xu, D. Saini, E. E. Hartwell, M. Pavicic, K. A. Sullivan, K. Xu, D. A. Jacobson, A multi-ancestry genetic study of pain intensity in 598,339 veterans. Nat. Med. 30, 2088 (2024). 38429522 10.1038/s41591-024-02839-5 PMC12105102

[3] C. Tanguay-Sabourin, M. Fillingim, G. V. Guglietti, A. Zare, M. Parisien, J. Norman, H. Sweatman, R. Da-Ano, E. Heikkala, PREVENT-AD Research Group, J. Perez, J. Karppinen, S. Villeneuve, S. J. Thompson, M. O. Martel, M. Roy, L. Diatchenko, E. Vachon-Presseau, A prognostic risk score for development and spread of chronic pain. Nat. Med. 29, 1821–1831 (2023). 37414898 10.1038/s41591-023-02430-4 PMC10353938

[4] R. J. Gatchel, Y. B. Peng, M. L. Peters, P. N. Fuchs, D. C. Turk, The biopsychosocial approach to chronic pain: Scientific advances and future directions. Psychol. Bull. 133, 581–624 (2007). 17592957 10.1037/0033-2909.133.4.581

[5] B. Kuehn, Chronic Pain Prevalence. JAMA 320, 1632 (2018). 10.1001/jama.2018.16009 30357307

[6] G. B. D. Diseases, C. Injuries, Global burden of 369 diseases and injuries in 204 countries and territories, 1990-2019: A systematic analysis for the Global Burden of Disease Study 2019. Lancet 396, 1204–1222 (2020). 33069326 10.1016/S0140-6736(20)30925-9 PMC7567026

[7] R. D. Treede, W. Rief, A. Barke, Q. Aziz, M. I. Bennett, R. Benoliel, M. Cohen, S. Evers, N. B. Finnerup, M. B. First, M. A. Giamberardino, S. Kaasa, B. Korwisi, E. Kosek, P. Lavand'homme, M. Nicholas, S. Perrot, J. Scholz, S. Schug, B. H. Smith, P. Svensson, J. W. S. Vlaeyen, S. J. Wang, Chronic pain as a symptom or a disease: the IASP Classification of Chronic Pain for the International Classification of Diseases (ICD-11). Pain 160, 19–27 (2019). 30586067 10.1097/j.pain.0000000000001384

[8] W. H. Meng, M. J. Adams, P. Reel, A. Rajendrakumar, Y. Huang, I. J. Deary, C. N. A. Palmer, A. M. McIntosh, B. H. Smith, Genetic correlations between pain phenotypes and depression and neuroticism. Eur. J. Hum. Genet. 28, 358–366 (2020). 31659249 10.1038/s41431-019-0530-2 PMC7028719

[9] L. X. Chen, C. E. Ashton-James, B. Y. Shi, M. R. Radojcic, D. B. Anderson, Y. J. Chen, D. B. Preen, J. L. Hopper, S. Li, M. Bui, P. R. Beckenkamp, N. K. Arden, P. H. Ferreira, H. X. Zhou, S. Q. Feng, M. L. Ferreira, Variability in the prevalence of depression among adults with chronic pain: UK Biobank analysis through clinical prediction models. BMC Med. 22, 167 (2024). 38637815 10.1186/s12916-024-03388-x PMC11027372

[10] L. Hu, Z. Z. Liu, Z. Y. Wang, C. X. Jia, X. C. Liu, Associations between pain and depressive symptoms: A longitudinal study of Chinese adolescents. J. Affect. Disord. 299, 675–681 (2022). 34953924 10.1016/j.jad.2021.12.095

[11] J. J. Wong, A. C. Tricco, P. Côté, C. Y. Liang, J. A. Lewis, Z. Bouck, L. C. Rosella, Association between depressive symptoms or depression and health outcomes for low back pain: A systematic review and meta-analysis. J. Gen. Intern. Med. 37, 1233–1246 (2022). 34383230 10.1007/s11606-021-07079-8 PMC8971223

[12] B. Stubbs, D. Vancampfort, N. Veronese, T. Thompson, M. Fornaro, P. Schofield, M. Solmi, J. Mugisha, A. F. Carvalho, A. Koyanagi, Depression and pain: primary data and meta-analysis among 237952 people across 47 low- and middle-income countries. Psychol. Med. 47, 2906–2917 (2017). 28637534 10.1017/S0033291717001477

[13] A. S. Rönnegård, C. Nowak, B. Äng, J. Ärnlöv, The association between short-term, chronic localized and chronic widespread pain and risk for cardiovascular disease in the UK Biobank. Eur. J. Prev. Cardiol. 29, 1994–2002 (2022). 35727954 10.1093/eurjpc/zwac127

[14] W. Maixner, R. B. Fillingim, D. A. Williams, S. B. Smith, G. D. Slade, Overlapping chronic pain conditions: Implications for diagnosis and classification. J. Pain 17, T93–T107 (2016). 27586833 10.1016/j.jpain.2016.06.002 PMC6193199

[15] K. W. Choi, M. B. Stein, K. M. Nishimi, T. Ge, J. R. I. Coleman, C. Y. Chen, A. Ratanatharathorn, A. B. Zheutlin, E. C. Dunn, 23andMe Research Team, Major Depressive Disorder Working Group of the Psychiatric Genomics Consortium, G. Breen, K. C. Koenen, J. W. Smoller, An exposure-wide and Mendelian randomization approach to identifying modifiable factors for the prevention of depression. Am. J. Psychiatry 177, 944–954 (2020). 32791893 10.1176/appi.ajp.2020.19111158 PMC9361193

[16] A. Schrepf, V. Phan, J. Q. Clemens, W. Maixner, D. Hanauer, D. A. Williams, ICD-10 codes for the study of chronic overlapping pain conditions in administrative databases. J. Pain 21, 59–70 (2020). 31154033 10.1016/j.jpain.2019.05.007 PMC8177096

[17] W. Zhao, L. Zhao, X. Chang, X. Lu, Y. Tu, Elevated dementia risk, cognitive decline, and hippocampal atrophy in multisite chronic pain. Proc. Natl. Acad. Sci. U.S.A. 120, e2215192120 (2023). 36802440 10.1073/pnas.2215192120 PMC9992778

[18] L. Zhao, J. Liu, W. Zhao, J. Chen, J. Fan, T. Ge, Y. Tu, Morphological and genetic decoding shows heterogeneous patterns of brain aging in chronic musculoskeletal pain. Nat. Mental Health 2, 435–449 (2024).

[19] R. R. Bhatt, E. Haddad, A. H. Zhu, P. M. Thompson, A. Gupta, E. A. Mayer, N. Jahanshad, Mapping brain structure variability in chronic pain: The role of widespreadness and pain type and its mediating relationship with suicide attempt. Biol. Psychiatry 95, 473–481 (2024). 37543299 10.1016/j.biopsych.2023.07.016 PMC10838358

[20] Y. Zhang, S. D. Chen, Y. T. Deng, J. You, X. Y. He, X. R. Wu, B. S. Wu, L. Yang, Y. R. Zhang, K. V. Kuo, J. F. Feng, W. Cheng, J. Suckling, A. D. Smith, J. T. Yu, Identifying modifiable factors and their joint effect on dementia risk in the UK Biobank. Nat. Hum. Behav. 7, 1185–1195 (2023). 37024724 10.1038/s41562-023-01585-x

[21] I. Lourida, E. Hannon, T. J. Littlejohns, K. M. Langa, E. Hypponen, E. Kuzma, D. J. Llewellyn, Association of lifestyle and genetic risk with incidence of dementia. JAMA 322, 430–437 (2019). 31302669 10.1001/jama.2019.9879 PMC6628594

[22] G. Ogliari, J. Ryg, K. Andersen-Ranberg, L. L. Scheel-Hincke, J. T. Collins, A. Cowley, C. Di Lorito, V. Booth, R. A. J. Smit, R. K. Akyea, N. Qureshi, D. A. Walsh, R. H. Harwood, T. Masud, Association between pain intensity and depressive symptoms in community-dwelling adults: Longitudinal findings from the Survey of Health, Ageing and Retirement in Europe (SHARE). Eur. Geriatr. Med. 14, 1111–1124 (2023). 37450107 10.1007/s41999-023-00835-5 PMC10587243

[23] B. Tang, W. Meng, S. Hägg, S. Burgess, X. Jiang, Reciprocal interaction between depression and pain: Results from a comprehensive bidirectional Mendelian randomization study and functional annotation analysis. Pain 163, e40–e48 (2022). 34924553 10.1097/j.pain.0000000000002305 PMC8675051

[24] W. Zhou, Y. Jin, Q. Meng, X. Zhu, T. Bai, Y. Tian, Y. Mao, L. Wang, W. Xie, H. Zhong, N. Zhang, M. H. Luo, W. Tao, H. Wang, J. Li, J. Li, B. S. Qiu, J. N. Zhou, X. Li, H. Xu, K. Wang, X. Zhang, Y. Liu, G. Richter-Levin, L. Xu, Z. Zhang, A neural circuit for comorbid depressive symptoms in chronic pain. Nat. Neurosci. 22, 1649–1658 (2019). 31451801 10.1038/s41593-019-0468-2

[25] Y. M. Bai, W. F. Chiou, T. P. Su, C. T. Li, M. H. Chen, Pro-inflammatory cytokine associated with somatic and pain symptoms in depression. J. Affect. Disord. 155, 28–34 (2014). 24176538 10.1016/j.jad.2013.10.019

[26] S. Cao, D. W. Fisher, T. Yu, H. Dong, The link between chronic pain and Alzheimer’s disease. J. Neuroinflammation 16, 204 (2019). 31694670 10.1186/s12974-019-1608-z PMC6836339

[27] R. R. Ji, A. Chamessian, Y. Q. Zhang, Pain regulation by non-neuronal cells and inflammation. Science 354, 572–577 (2016). 27811267 10.1126/science.aaf8924 PMC5488328

[28] R. R. Ji, Z. Z. Xu, Y. J. Gao, Emerging targets in neuroinflammation-driven chronic pain. Nat. Rev. Drug Discov. 13, 533–548 (2014). 24948120 10.1038/nrd4334 PMC4228377

[29] A. H. Miller, C. L. Raison, The role of inflammation in depression: From evolutionary imperative to modern treatment target. Nat. Rev. Immunol. 16, 22–34 (2016). 26711676 10.1038/nri.2015.5 PMC5542678

[30] R. Dantzer, J. C. O'Connor, G. G. Freund, R. W. Johnson, K. W. Kelley, From inflammation to sickness and depression: When the immune system subjugates the brain. Nat. Rev. Neurosci. 9, 46–56 (2008). 18073775 10.1038/nrn2297 PMC2919277

[31] P. Frank, M. Jokela, G. D. Batty, D. Cadar, A. Steptoe, M. Kivimäki, Association between systemic inflammation and individual symptoms of depression: A pooled analysis of 15 population-based cohort studies. Am. J. Psychiatry 178, 1107–1118 (2021). 34645276 10.1176/appi.ajp.2021.20121776

[32] N. M. Davies, M. V. Holmes, G. Davey Smith, Reading Mendelian randomisation studies: A guide, glossary, and checklist for clinicians. BMJ 362, k601 (2018). 30002074 10.1136/bmj.k601 PMC6041728

[33] M. Caliendo, S. Kopeinig, Some practical guidance for the implementation of propensity score matching. J. Econ. Surv. 22, 31–72 (2008).

[34] Y. J. Qiu, Y. J. Ma, X. B. Huang, Bidirectional relationship between body pain and depressive symptoms: A pooled analysis of two national aging cohort studies. Front. Psych. 13, 881779 (2022). 10.3389/fpsyt.2022.881779 PMC9086823 35558432

[35] D. B. Carr, L. C. Goudas, Acute pain. Lancet 353, 2051–2058 (1999). 10376632 10.1016/S0140-6736(99)03313-9

[36] J. Sarris, R. Thomson, F. Hargraves, M. Eaton, M. de Manincor, N. Veronese, M. Solmi, B. Stubbs, A. R. Yung, J. Firth, Multiple lifestyle factors and depressed mood: A cross-sectional and longitudinal analysis of the UK Biobank (N = 84,860). BMC Med. 18, 354 (2020). 33176802 10.1186/s12916-020-01813-5 PMC7661271

[37] Y. Zhao, L. Yang, B. J. Sahakian, C. Langley, W. Zhang, K. Kuo, Z. Li, Y. Gan, Y. Li, Y. Zhao, The brain structure, immunometabolic and genetic mechanisms underlying the association between lifestyle and depression. Nat. Mental Health 1, 736–750 (2023).

[38] S. Campbell, G. Macqueen, The role of the hippocampus in the pathophysiology of major depression. J. Psychiatry Neurosci. 29, 417–426 (2004). 15644983 PMC524959

[39] S. Khoury, M. Parisien, S. J. Thompson, E. Vachon-Presseau, M. Roy, A. E. Martinsen, B. S. Winsvold, H. A.-I. Pain, I. P. Mundal, J. A. Zwart, A. Kania, J. S. Mogil, L. Diatchenko, Genome-wide analysis identifies impaired axonogenesis in chronic overlapping pain conditions. Brain 145, 1111–1123 (2022). 34788396 10.1093/brain/awab359

[40] Y. Chen, Y. Sun, L. Wang, K. Xu, D. W. Wang, Genetic insights into associations of multisite chronic pain with common diseases and biomarkers using data from the UK Biobank. Br. J. Anaesth. 132, 372–382 (2024). 38104003 10.1016/j.bja.2023.11.007

[41] J. H. Zhu, N. N. Wang, H. P. Liu, H. Jiang, B. Y. Cai, D. W. Chen, Y. J. Li, Multisite chronic pain as a causal risk factor for coronary artery disease: Findings from Mendelian randomization. Pain 164, E135–E143 (2023). 35916731 10.1097/j.pain.0000000000002732

[42] Y. L. Wang, S. Genon, D. B. Dong, F. Zhou, C. Y. Li, D. H. Yu, K. Yuan, Q. H. He, J. Qiu, T. Y. Feng, H. Chen, X. Lei, Covariance patterns between sleep health domains and distributed intrinsic functional connectivity. Nat. Commun. 14, 7133 (2023). 37932259 10.1038/s41467-023-42945-5 PMC10628193

[43] L. Jiao, P. N. Mitrou, J. Reedy, B. I. Graubard, A. R. Hollenbeck, A. Schatzkin, R. Stolzenberg-Solomon, A combined healthy lifestyle score and risk of pancreatic cancer in a large cohort study. Arch. Intern. Med. 169, 764–770 (2009). 19398688 10.1001/archinternmed.2009.46 PMC3498842

[44] Y. Kamaleri, B. Natvig, C. M. Ihlebaek, J. S. Benth, D. Bruusgaard, Number of pain sites is associated with demographic, lifestyle, and health-related factors in the general population. Eur. J. Pain 12, 742–748 (2008). 18160318 10.1016/j.ejpain.2007.11.005

[45] J. L. Riley, Y. Cruz-Almeida, T. L. Glover, C. D. King, B. R. Goodin, K. T. Sibille, E. J. Bartley, M. S. Herbert, A. Sotolongo, B. J. Fessler, D. T. Redden, R. Staud, L. A. Bradley, R. B. Fillingim, Age and race effects on pain sensitivity and modulation among middle-aged and older adults. J. Pain 15, 272–282 (2014). 24239561 10.1016/j.jpain.2013.10.015 PMC4005289

[46] L. Ji, X. Qiao, Y. Jin, H. Si, X. Liu, C. Wang, Age differences in the relationship between frailty and depression among community-dwelling older adults. Geriatr. Nurs. 41, 485–489 (2020). 32087977 10.1016/j.gerinurse.2020.01.021

[47] M. L. Loggia, D. B. Chonde, O. Akeju, G. Arabasz, C. Catana, R. R. Edwards, E. Hill, S. Hsu, D. Izquierdo-Garcia, R. R. Ji, M. Riley, A. D. Wasan, N. R. Zurcher, D. S. Albrecht, M. G. Vangel, B. R. Rosen, V. Napadow, J. M. Hooker, Evidence for brain glial activation in chronic pain patients. Brain 138, 604–615 (2015). 25582579 10.1093/brain/awu377 PMC4339770

[48] A. C. P. Campos, G. F. Antunes, M. Matsumoto, R. L. Pagano, R. C. R. Martinez, Neuroinflammation, pain and depression: An overview of the main findings. Front. Psychol. 11, 1825 (2020). 32849076 10.3389/fpsyg.2020.01825 PMC7412934

[49] G. Chen, Y. Q. Zhang, Y. J. Qadri, C. N. Serhan, R. R. Ji, Microglia in pain: Detrimental and protective roles in pathogenesis and resolution of pain. Neuron 100, 1292–1311 (2018). 30571942 10.1016/j.neuron.2018.11.009 PMC6312407

[50] J. A. Williams, S. Burgess, J. Suckling, P. A. Lalousis, F. Batool, S. L. Griffiths, E. Palmer, A. Karwath, A. Barsky, G. V. Gkoutos, S. Wood, N. M. Barnes, A. S. David, G. Donohoe, J. C. Neill, B. Deakin, G. M. Khandaker, R. Upthegrove, PIMS Collaboration, Inflammation and brain structure in schizophrenia and other neuropsychiatric disorders: A mendelian randomization study. JAMA Psychiatry 79, 498–507 (2022). 35353173 10.1001/jamapsychiatry.2022.0407 PMC8968718

[51] M. T. Heneka, R. M. McManus, E. Latz, Inflammasome signalling in brain function and neurodegenerative disease. Nat. Rev. Neurosci. 19, 610–621 (2018). 30206330 10.1038/s41583-018-0055-7

[52] M. Cen, L. Song, X. Fu, X. Gao, Q. Zuo, J. Wu, Associations between metabolic syndrome and anxiety, and the mediating role of inflammation: Findings from the UK Biobank. Brain Behav. Immun. 116, 1–9 (2024). 37984624 10.1016/j.bbi.2023.11.019

[53] Y. Li, B. J. Sahakian, J. Kang, C. Langley, W. Zhang, C. Xie, S. Xiang, J. Yu, W. Cheng, J. Feng, The brain structure and genetic mechanisms underlying the nonlinear association between sleep duration, cognition and mental health. Nat. Aging 2, 425–437 (2022). 37118065 10.1038/s43587-022-00210-2

[54] J. Firth, M. Solmi, R. E. Wootton, D. Vancampfort, F. B. Schuch, E. Hoare, S. Gilbody, J. Torous, S. B. Teasdale, S. E. Jackson, L. Smith, M. Eaton, F. N. Jacka, N. Veronese, W. Marx, G. Ashdown-Franks, D. Siskind, J. Sarris, S. Rosenbaum, A. F. Carvalho, B. Stubbs, A meta-review of “lifestyle psychiatry”: The role of exercise, smoking, diet and sleep in the prevention and treatment of mental disorders. World Psychiatry 19, 360–380 (2020). 32931092 10.1002/wps.20773 PMC7491615

[55] Y.-R. Zhang, Y.-T. Deng, Y.-Z. Li, R.-Q. Zhang, K. Kuo, Y.-J. Ge, B.-S. Wu, W. Zhang, A. D. Smith, J. Suckling, Personality traits and brain health: A large prospective cohort study. Nat. Mental Health 1, 722–735 (2023).

[56] B. H. Smith, N. Torrance, Management of chronic pain in primary care. Curr. Opin. Support. Palliat. Care 5, 137–142 (2011). 21415754 10.1097/SPC.0b013e328345a3ec

[57] K. Zorina-Lichtenwalter, C. I. Bango, L. Van Oudenhove, M. Ceko, M. A. Lindquist, A. D. Grotzinger, M. C. Keller, N. P. Friedman, T. D. Wager, Genetic risk shared across 24 chronic pain conditions: identification and characterization with genomic structural equation modeling. Pain 164, 2239–2252 (2023). 37219871 10.1097/j.pain.0000000000002922 PMC10524350

[58] S. E. E. Mills, K. P. Nicolson, B. H. Smith, Chronic pain: A review of its epidemiology and associated factors in population-based studies. Br. J. Anaesth. 123, e273–e283 (2019). 31079836 10.1016/j.bja.2019.03.023 PMC6676152

[59] C. Sudlow, J. Gallacher, N. Allen, V. Beral, P. Burton, J. Danesh, P. Downey, P. Elliott, J. Green, M. Landray, UK biobank: an open access resource for identifying the causes of a wide range of complex diseases of middle and old age. PLOS Med. 12, e1001779 (2015). 25826379 10.1371/journal.pmed.1001779 PMC4380465

[60] K. J. A. Johnston, M. J. Adams, B. I. Nicholl, J. Ward, R. J. Strawbridge, A. Ferguson, A. M. McIntosh, M. E. S. Bailey, D. J. Smith, Genome-wide association study of multisite chronic pain in UK Biobank. PLOS Genet. 15, e1008164 (2019). 31194737 10.1371/journal.pgen.1008164 PMC6592570

[61] B. I. Nicholl, D. Mackay, B. Cullen, D. J. Martin, Z. Ul-Haq, F. S. Mair, J. Evans, A. M. McIntosh, J. Gallagher, B. Roberts, I. J. Deary, J. P. Pell, D. J. Smith, Chronic multisite pain in major depression and bipolar disorder: Cross-sectional study of 149,611 participants in UK Biobank. BMC Psychiatry 14, 350 (2014). 25490859 10.1186/s12888-014-0350-4 PMC4297369

[62] T. Yang, J. Wang, J. Huang, F. J. Kelly, G. Li, Long-term exposure to multiple ambient air pollutants and association with incident depression and anxiety. JAMA Psychiatry 80, 305–313 (2023). 36723924 10.1001/jamapsychiatry.2022.4812 PMC10077109

[63] K. T. Watson, J. F. Simard, V. W. Henderson, L. Nutkiewicz, F. Lamers, C. Nasca, N. Rasgon, B. Penninx, Incident major depressive disorder predicted by three measures of insulin resistance: A dutch cohort study. Am. J. Psychiatry 178, 914–920 (2021). 34551583 10.1176/appi.ajp.2021.20101479

[64] A. Dregan, L. Rayner, K. A. S. Davis, I. Bakolis, J. A. De La Torre, J. Das-Munshi, S. L. Hatch, R. Stewart, M. Hotopf, Associations between depression, arterial stiffness, and metabolic syndrome among adults in the UK Biobank population study: A mediation analysis. JAMA Psychiatry 77, 598–606 (2020). 31995135 10.1001/jamapsychiatry.2019.4712 PMC6990710

[65] H. M. E. Foster, C. A. Celis-Morales, B. I. Nicholl, F. Petermann-Rocha, J. P. Pell, J. M. R. Gill, C. A. O'Donnell, F. S. Mair, The effect of socioeconomic deprivation on the association between an extended measurement of unhealthy lifestyle factors and health outcomes: A prospective analysis of the UK Biobank cohort. Lancet Public Health 3, e576–e585 (2018). 30467019 10.1016/S2468-2667(18)30200-7

[66] R. Jiang, S. Noble, M. Rosenblatt, W. Dai, J. Ye, S. Liu, S. Qi, V. D. Calhoun, J. Sui, D. Scheinost, The brain structure, inflammatory, and genetic mechanisms mediate the association between physical frailty and depression. Nat. Commun. 15, 4411 (2024). 38782943 10.1038/s41467-024-48827-8 PMC11116547

[67] Y. Wang, B. Su, J. Xie, C. Garcia-Rizo, D. Prieto-Alhambra, Long-term risk of psychiatric disorder and psychotropic prescription after SARS-CoV-2 infection among UK general population. Nat. Hum. Behav. 8, 1076–1087 (2024). 38514769 10.1038/s41562-024-01853-4 PMC11199144

[68] D. E. Ho, K. Imai, G. King, E. A. Stuart, MatchIt: Nonparametric preprocessing for parametric causal inference. J. Stat. Softw. 42, 1–28 (2011).

[69] S. R. Cox, D. M. Lyall, S. J. Ritchie, M. E. Bastin, M. A. Harris, C. R. Buchanan, C. Fawns-Ritchie, M. C. Barbu, L. De Nooij, L. M. Reus, C. Alloza, X. Y. Shen, E. Neilson, H. L. Alderson, S. Hunter, D. C. Liewald, H. C. Whalley, A. M. McIntosh, S. M. Lawrie, J. P. Pell, E. M. Tucker-Drob, J. M. Wardlaw, C. R. Gale, I. J. Deary, Associations between vascular risk factors and brain MRI indices in UK Biobank. Eur. Heart J. 40, 2290–2300 (2019). 30854560 10.1093/eurheartj/ehz100 PMC6642726

[70] G. Hemani, J. Zheng, B. Elsworth, K. H. Wade, V. Haberland, D. Baird, C. Laurin, S. Burgess, J. Bowden, R. Langdon, V. Y. Tan, J. Yarmolinsky, H. A. Shihab, N. J. Timpson, D. M. Evans, C. Relton, R. M. Martin, G. Davey Smith, T. R. Gaunt, P. C. Haycock, The MR-Base platform supports systematic causal inference across the human phenome. eLife 7, e34408 (2018). 29846171 10.7554/eLife.34408 PMC5976434

[71] T. D. Als, M. I. Kurki, J. Grove, G. Voloudakis, K. Therrien, E. Tasanko, T. T. Nielsen, J. Naamanka, K. Veerapen, D. F. Levey, J. Bendl, J. Bybjerg-Grauholm, B. Zeng, D. Demontis, A. Rosengren, G. Athanasiadis, M. Baekved-Hansen, P. Qvist, G. Bragi Walters, T. Thorgeirsson, H. Stefansson, K. L. Musliner, V. M. Rajagopal, L. Farajzadeh, J. Thirstrup, B. J. Vilhjalmsson, J. J. McGrath, M. Mattheisen, S. Meier, E. Agerbo, K. Stefansson, M. Nordentoft, T. Werge, D. M. Hougaard, P. B. Mortensen, M. B. Stein, J. Gelernter, I. Hovatta, P. Roussos, M. J. Daly, O. Mors, A. Palotie, A. D. Borglum, Depression pathophysiology, risk prediction of recurrence and comorbid psychiatric disorders using genome-wide analyses. Nat. Med. 29, 1832–1844 (2023). 37464041 10.1038/s41591-023-02352-1 PMC10839245

[72] N. R. Wray, S. Ripke, M. Mattheisen, M. Trzaskowski, E. M. Byrne, A. Abdellaoui, M. J. Adams, E. Agerbo, T. M. Air, T. M. F. Andlauer, S. A. Bacanu, M. Baekvad-Hansen, A. F. T. Beekman, T. B. Bigdeli, E. B. Binder, D. R. H. Blackwood, J. Bryois, H. N. Buttenschon, J. Bybjerg-Grauholm, N. Cai, E. Castelao, J. H. Christensen, T. K. Clarke, J. I. R. Coleman, L. Colodro-Conde, B. Couvy-Duchesne, N. Craddock, G. E. Crawford, C. A. Crowley, H. S. Dashti, G. Davies, I. J. Deary, F. Degenhardt, E. M. Derks, N. Direk, C. V. Dolan, E. C. Dunn, T. C. Eley, N. Eriksson, V. Escott-Price, F. H. F. Kiadeh, H. K. Finucane, A. J. Forstner, J. Frank, H. A. Gaspar, M. Gill, P. Giusti-Rodriguez, F. S. Goes, S. D. Gordon, J. Grove, L. S. Hall, E. Hannon, C. S. Hansen, T. F. Hansen, S. Herms, I. B. Hickie, P. Hoffmann, G. Homuth, C. Horn, J. J. Hottenga, D. M. Hougaard, M. Hu, C. L. Hyde, M. Ising, R. Jansen, F. Jin, E. Jorgenson, J. A. Knowles, I. S. Kohane, J. Kraft, W. W. Kretzschmar, J. Krogh, Z. Kutalik, J. M. Lane, Y. Li, Y. Li, P. A. Lind, X. Liu, L. Lu, D. J. MacIntyre, D. F. MacKinnon, R. M. Maier, W. Maier, J. Marchini, H. Mbarek, P. McGrath, P. McGuffin, S. E. Medland, D. Mehta, C. M. Middeldorp, E. Mihailov, Y. Milaneschi, L. Milani, J. Mill, F. M. Mondimore, G. W. Montgomery, S. Mostafavi, N. Mullins, M. Nauck, B. Ng, M. G. Nivard, D. R. Nyholt, P. F. O'Reilly, H. Oskarsson, M. J. Owen, J. N. Painter, C. B. Pedersen, M. G. Pedersen, R. E. Peterson, E. Pettersson, W. J. Peyrot, G. Pistis, D. Posthuma, S. M. Purcell, J. A. Quiroz, P. Qvist, J. P. Rice, B. P. Riley, M. Rivera, S. S. Mirza, R. Saxena, R. Schoevers, E. C. Schulte, L. Shen, J. Shi, S. I. Shyn, E. Sigurdsson, G. B. C. Sinnamon, J. H. Smit, D. J. Smith, H. Stefansson, S. Steinberg, C. A. Stockmeier, F. Streit, J. Strohmaier, K. E. Tansey, H. Teismann, A. Teumer, W. Thompson, P. A. Thomson, T. E. Thorgeirsson, C. Tian, M. Traylor, J. Treutlein, V. Trubetskoy, A. G. Uitterlinden, D. Umbricht, S. Van der Auwera, A. M. van Hemert, A. Viktorin, P. M. Visscher, Y. Wang, B. T. Webb, S. M. Weinsheimer, J. Wellmann, G. Willemsen, S. H. Witt, Y. Wu, H. S. Xi, J. Yang, F. Zhang, eQtlgen, 23andMe, V. Arolt, B. T. Baune, K. Berger, D. I. Boomsma, S. Cichon, U. Dannlowski, E. C. J. de Geus, J. R. DePaulo, E. Domenici, K. Domschke, T. Esko, H. J. Grabe, S. P. Hamilton, C. Hayward, A. C. Heath, D. A. Hinds, K. S. Kendler, S. Kloiber, G. Lewis, Q. S. Li, S. Lucae, P. F. A. Madden, P. K. Magnusson, N. G. Martin, A. M. McIntosh, A. Metspalu, O. Mors, P. B. Mortensen, B. Muller-Myhsok, M. Nordentoft, M. M. Nothen, M. C. O'Donovan, S. A. Paciga, N. L. Pedersen, B. Penninx, R. H. Perlis, D. J. Porteous, J. B. Potash, M. Preisig, M. Rietschel, C. Schaefer, T. G. Schulze, J. W. Smoller, K. Stefansson, H. Tiemeier, R. Uher, H. Volzke, M. M. Weissman, T. Werge, A. R. Winslow, C. M. Lewis, D. F. Levinson, G. Breen, A. D. Borglum, P. F. Sullivan, Major Depressive Disorder Working Group of the Psychiatric Genomics Consortium, Genome-wide association analyses identify 44 risk variants and refine the genetic architecture of major depression. Nat. Genet. 50, 668–681 (2018). 29700475 10.1038/s41588-018-0090-3 PMC5934326

[73] R. Jiang, S. Noble, J. Sui, K. Yoo, M. Rosenblatt, C. Horien, S. Qi, Q. Liang, H. Sun, V. D. Calhoun, D. Scheinost, Associations of physical frailty with health outcomes and brain structure in 483 033 middle-aged and older adults: A population-based study from the UK Biobank. Lancet Digit Health 5, e350–e359 (2023). 37061351 10.1016/S2589-7500(23)00043-2 PMC10257912

[74] R. L. Grant, Converting an odds ratio to a range of plausible relative risks for better communication of research findings. BMJ 348, f7450 (2014). 24464277 10.1136/bmj.f7450

[75] Y. T. Deng, Y. Z. Li, S. Y. Huang, Y. N. Ou, W. Zhang, S. D. Chen, Y. R. Zhang, L. Yang, Q. Dong, J. F. Feng, J. Suckling, A. D. Smith, W. Cheng, J. T. Yu, Association of life course adiposity with risk of incident dementia: A prospective cohort study of 322,336 participants. Mol. Psychiatry 27, 3385–3395 (2022). 35538193 10.1038/s41380-022-01604-9

