# Supplementary material for: The inflammatory and genetic mechanisms underlying the cumulative effect of co-occurring pain conditions on depression
Source: Sci Adv. 2025 Apr 2;11(14):eadt1083. doi: 10.1126/sciadv.adt1083 (PMC11964001; doi:10.1126/sciadv.adt1083)
Supplement: Supplementary file 1 — Figs. S1 to S14 Tables S1 to S14 [file sciadv.adt1083_sm.pdf]

Science Advances
AAAS

Supplementary Materials for

The inflammatory and genetic mechanisms underlying the cumulative effect of co-occurring pain conditions on depression

Rongtao Jiang *et al.*

Corresponding author: Rongtao Jiang, rongtao.jiang@yale.edu; Yunhe Wang, yunhe.wang@ndph.ox.ac.uk; Jing Sui, jsui@bnu.edu.cn

Sci. Adv. **11**, eadt1083 (2025)
DOI: 10.1126/sciadv.adt1083

This PDF file includes:

Figs. S1 to S14
Tables S1 to S14

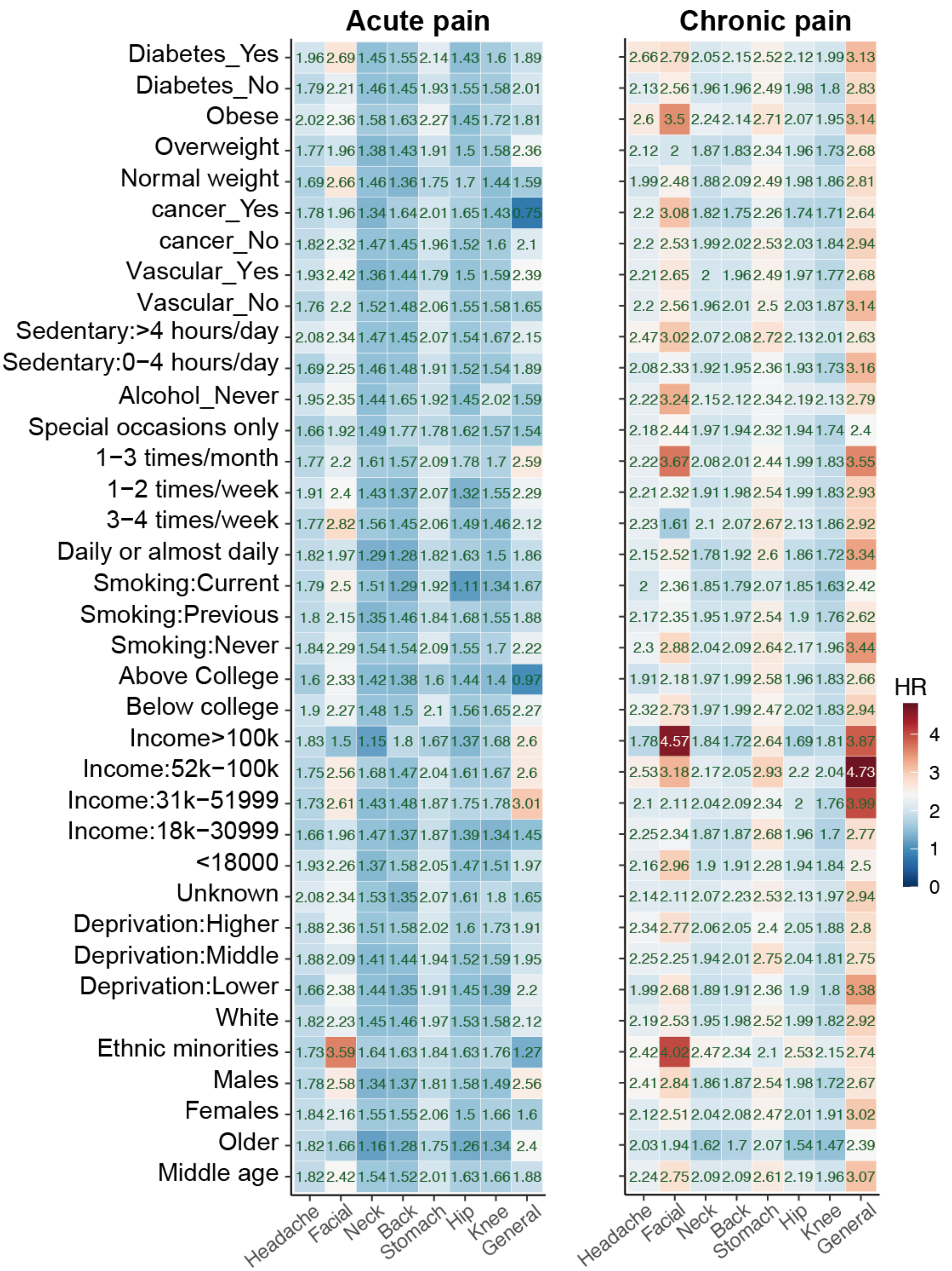

Fig. S1. Associations between acute or chronic pain at eight body sites and incident depression by subgroups.

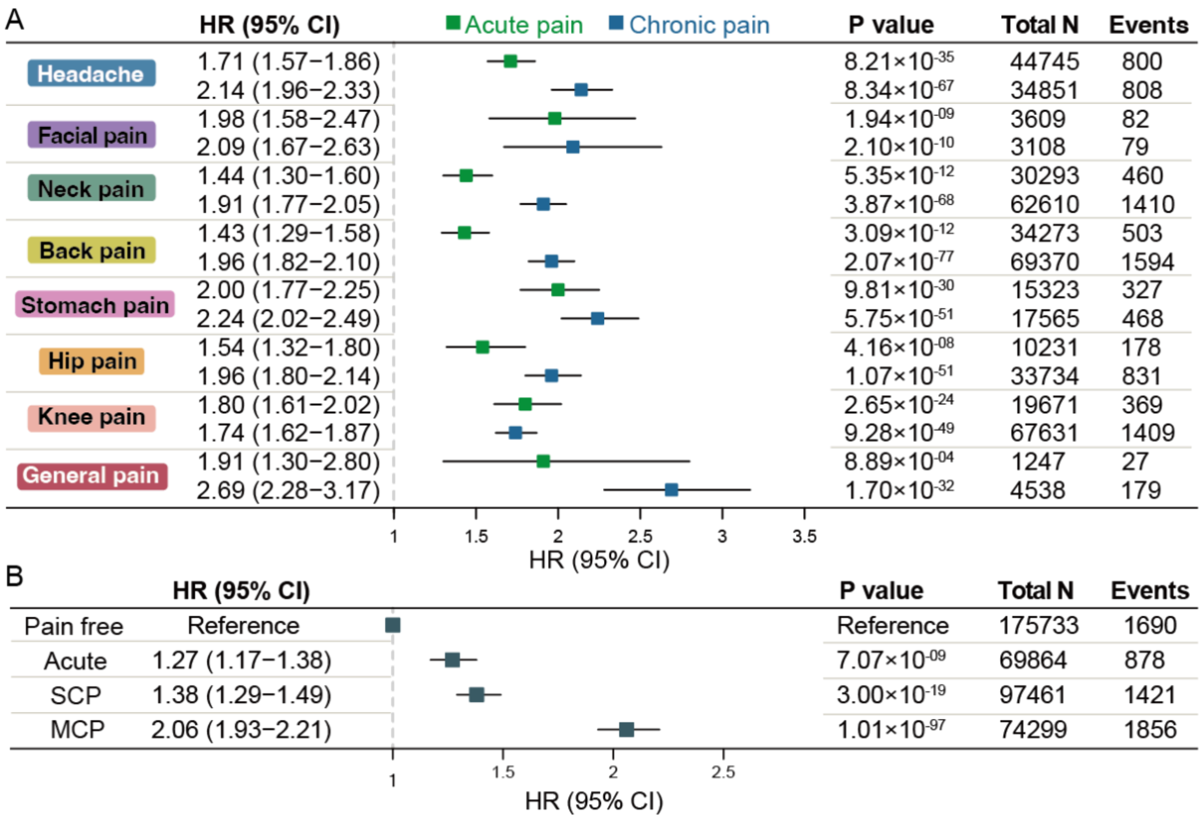

Fig. S2. Associations between pain conditions and depression incidence using a 10-year landmark analysis. In this sensitivity analysis, we excluded participants experiencing events within the 10-year follow-up period. This analysis included a total of 426,906 participants for the analysis of pain at eight body sites. After excluding participants reporting general pain, 421,819 participants were used for calculating the number of coexisting pain sites.

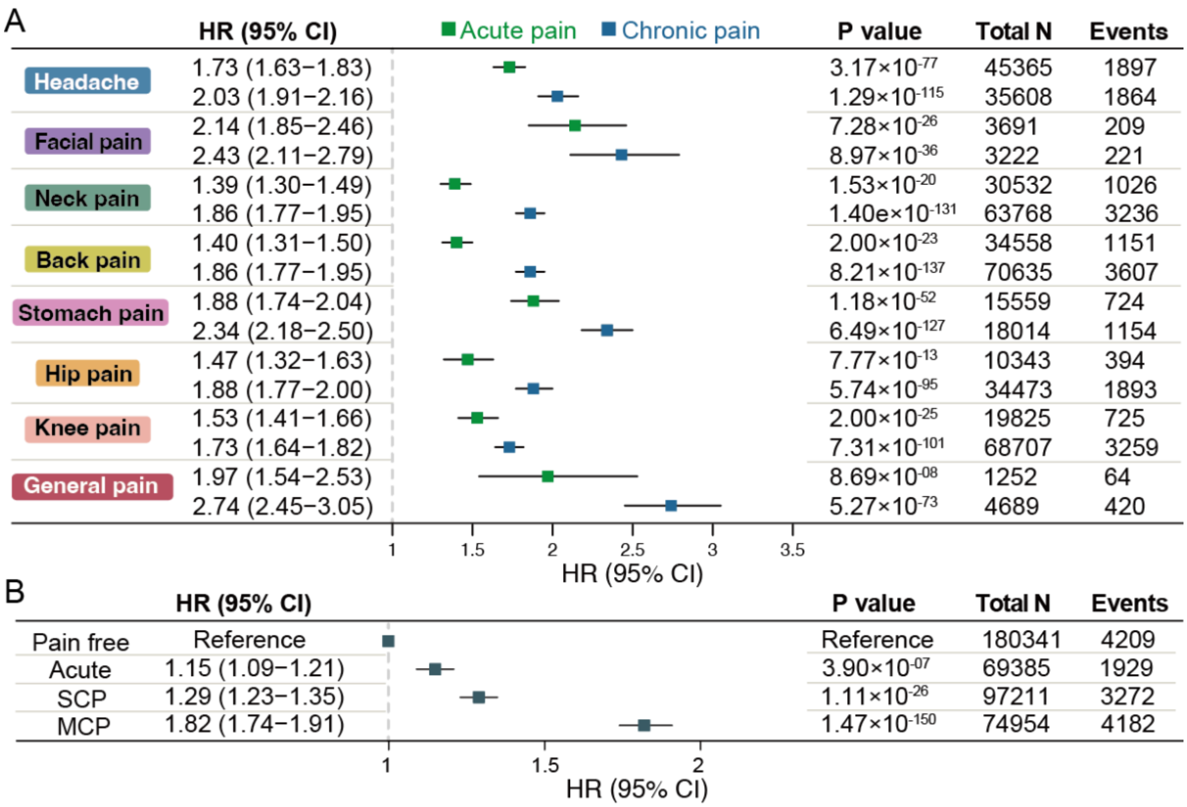

Fig. S3. Associations between pain conditions and depression incidence after additionally adjusting for the effect of taking pain medications including aspirin, paracetamol, and ibuprofen.

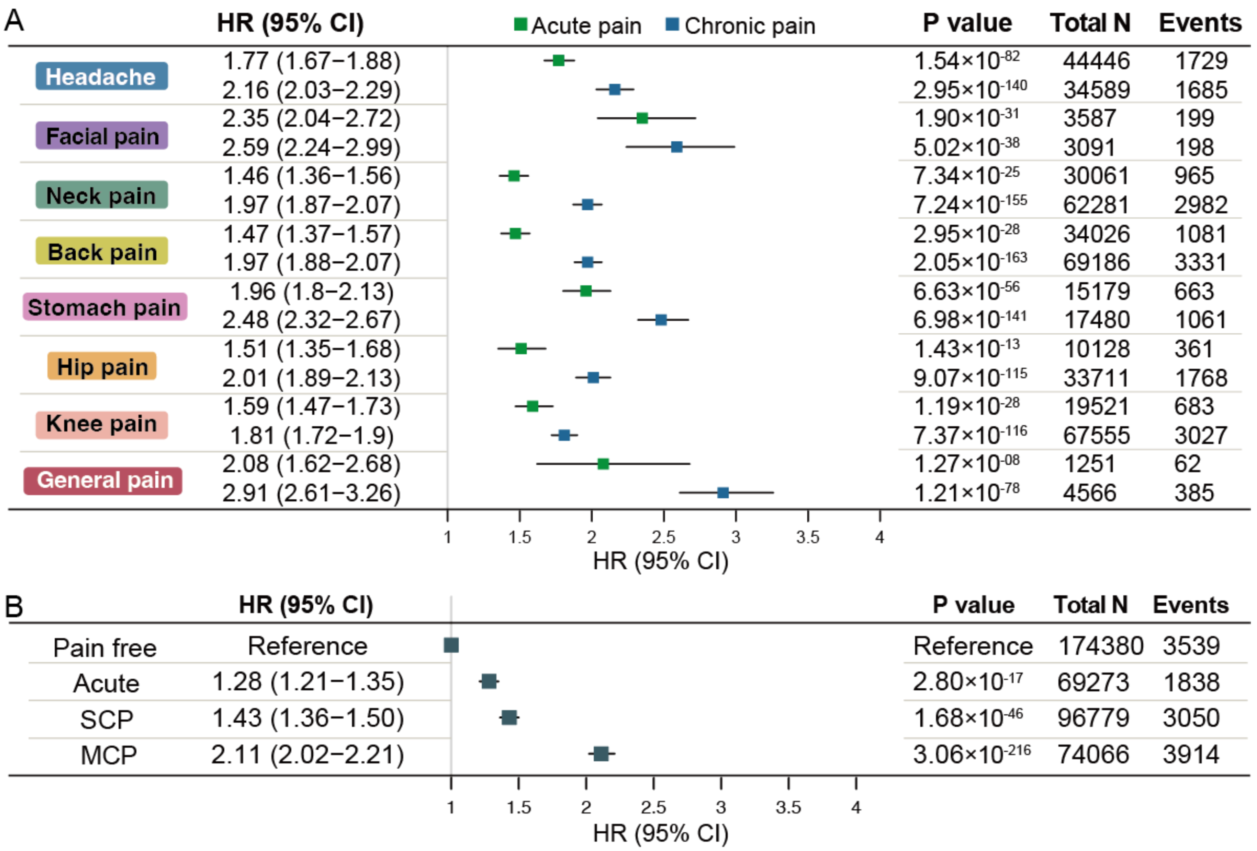

Fig. S4. Associations between pain conditions and depression incidence after excluding participants having anxiety at baseline (anxiety was defined as ICD-10 codes F40 or F41).

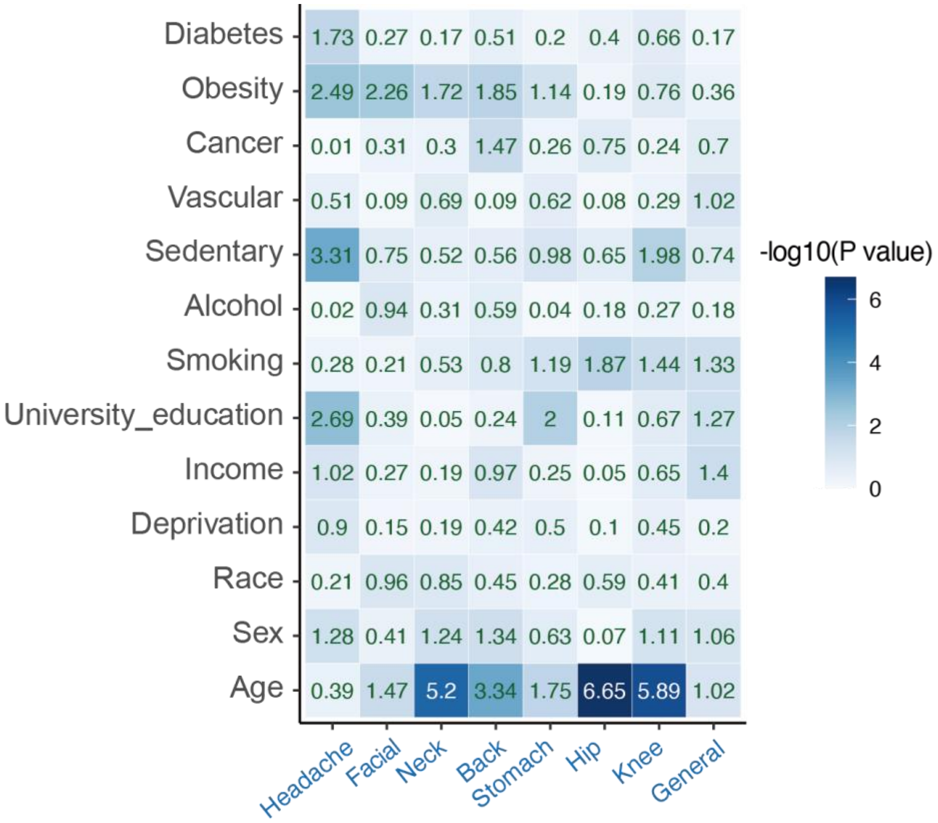

Fig. S5. Interaction analyses supported a significantly modifying effect of age on the association of pain at neck, back, hip, and knee with the risk of depression (Bonferroni-corrected P threshold <0.05/13 for 13 tests), but not for the other covariates.

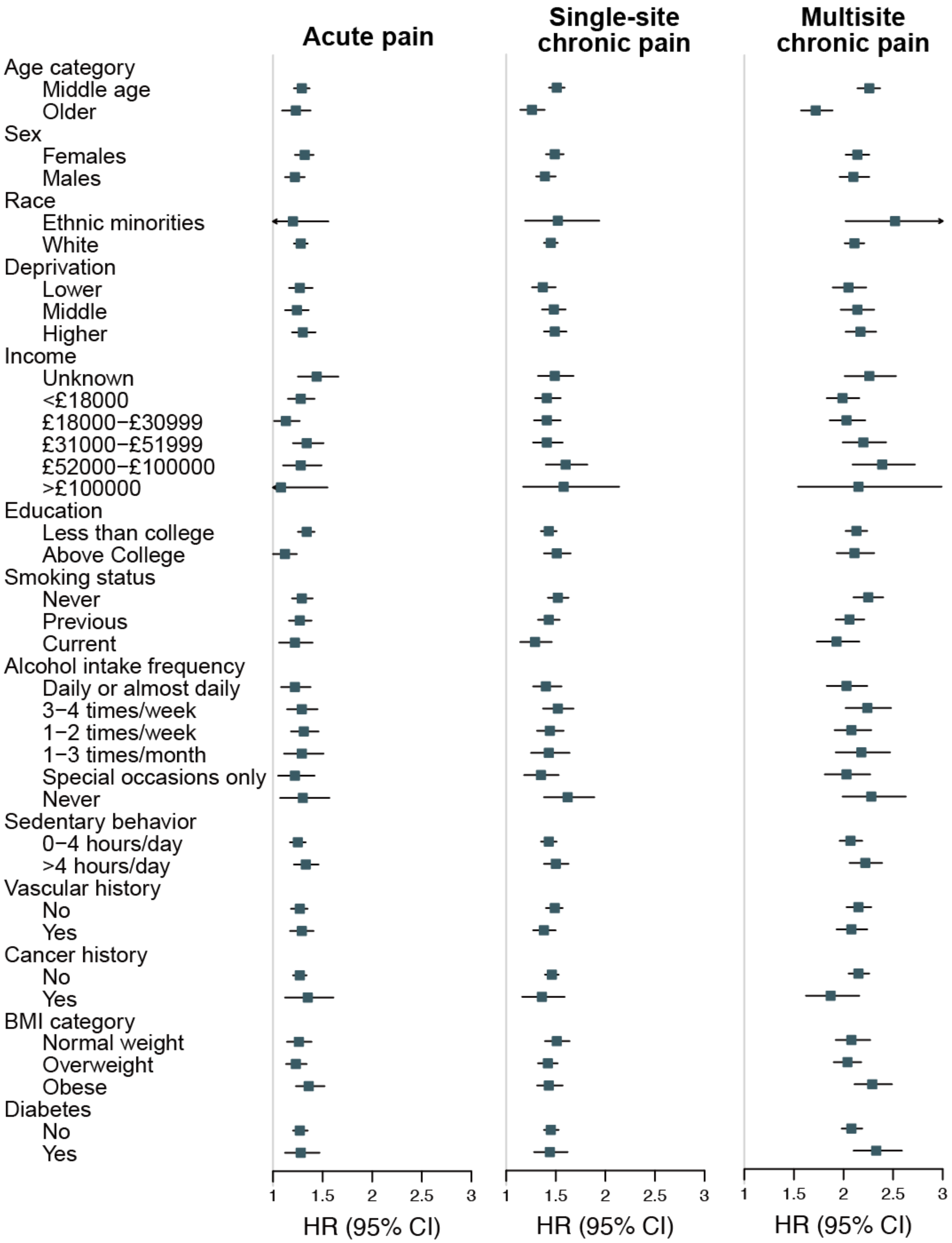

Fig. S6. Associations of acute pain, SCP, and MCP with the risk of depression in subgroups.

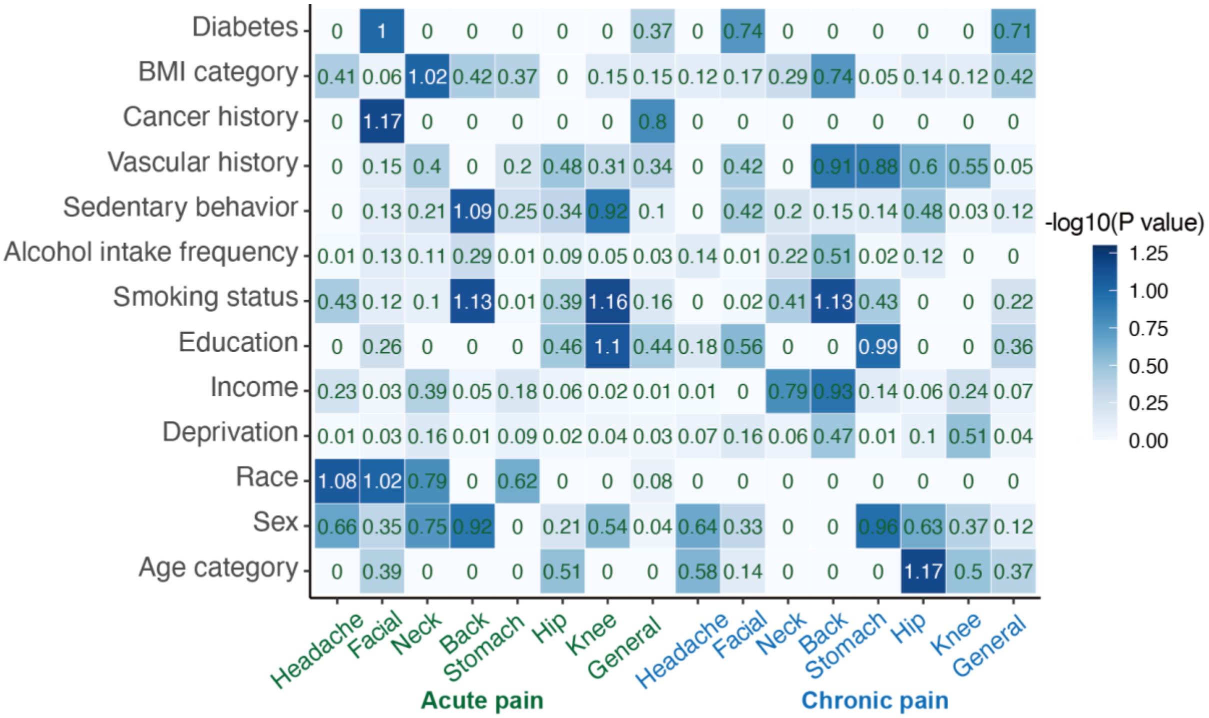

Fig. S7: Difference in covariates between participants with pain at a specific body site and matched pain-free participants. No significant difference was observed for any covariates.

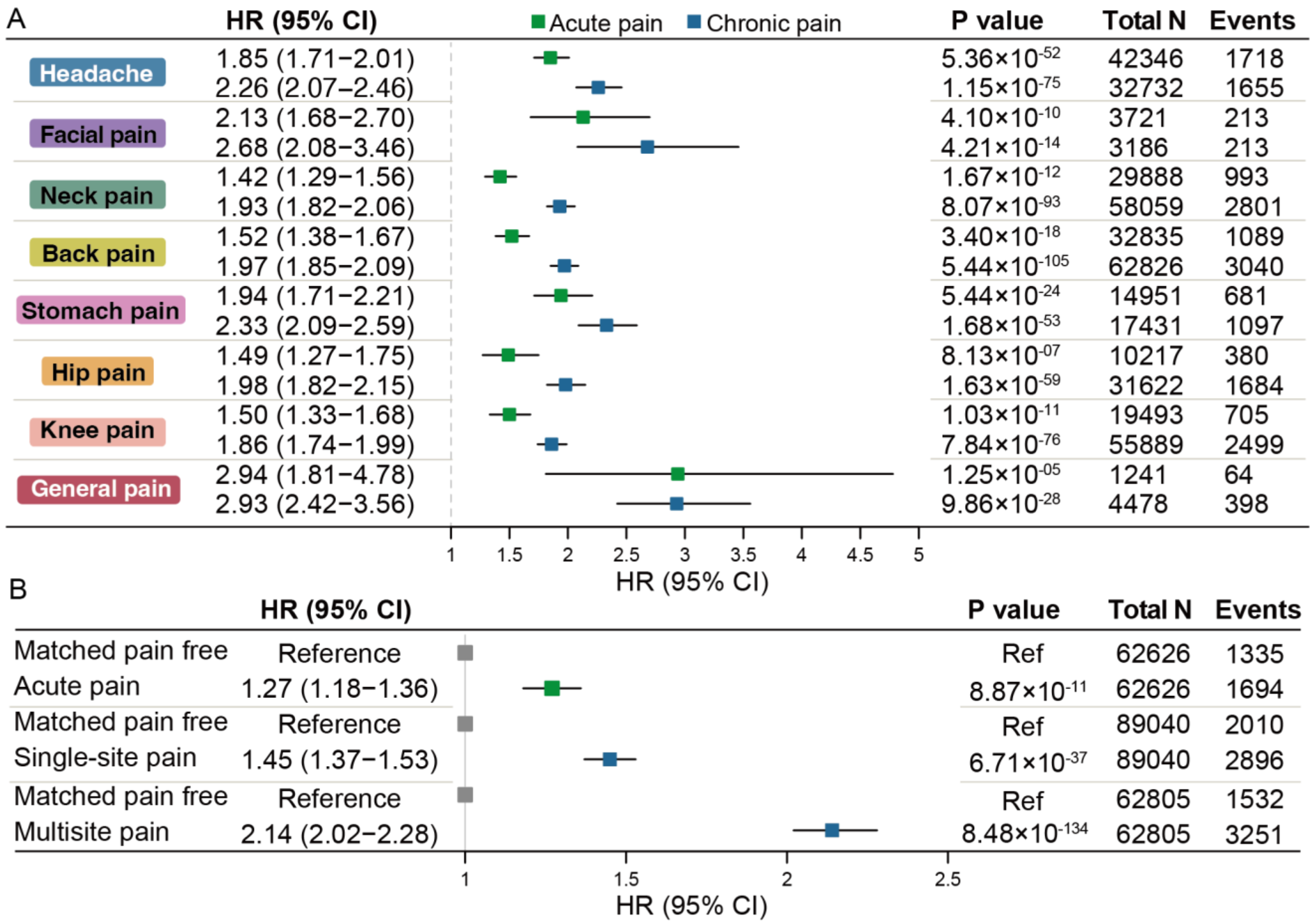

Fig. S8: Associations between pain conditions and incident depression based on propensity-matched participants.

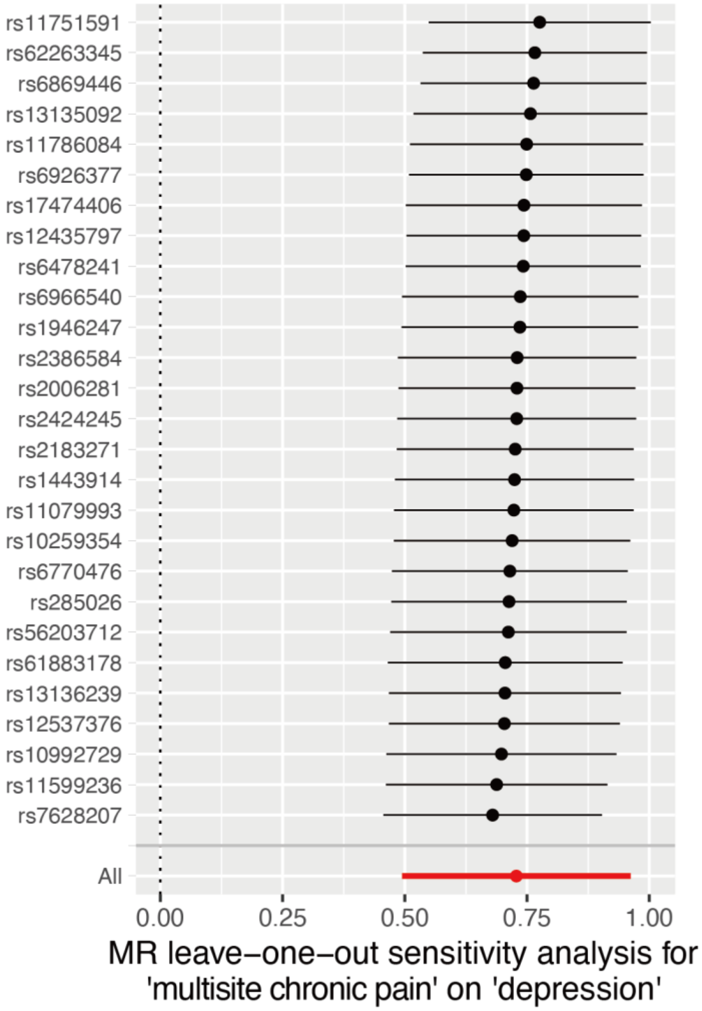

Fig. S9. Forest plot of leave-one-SNP-out sensitivity analysis. Dots: estimated beta coefficients; Horizontal lines: 95% CI.

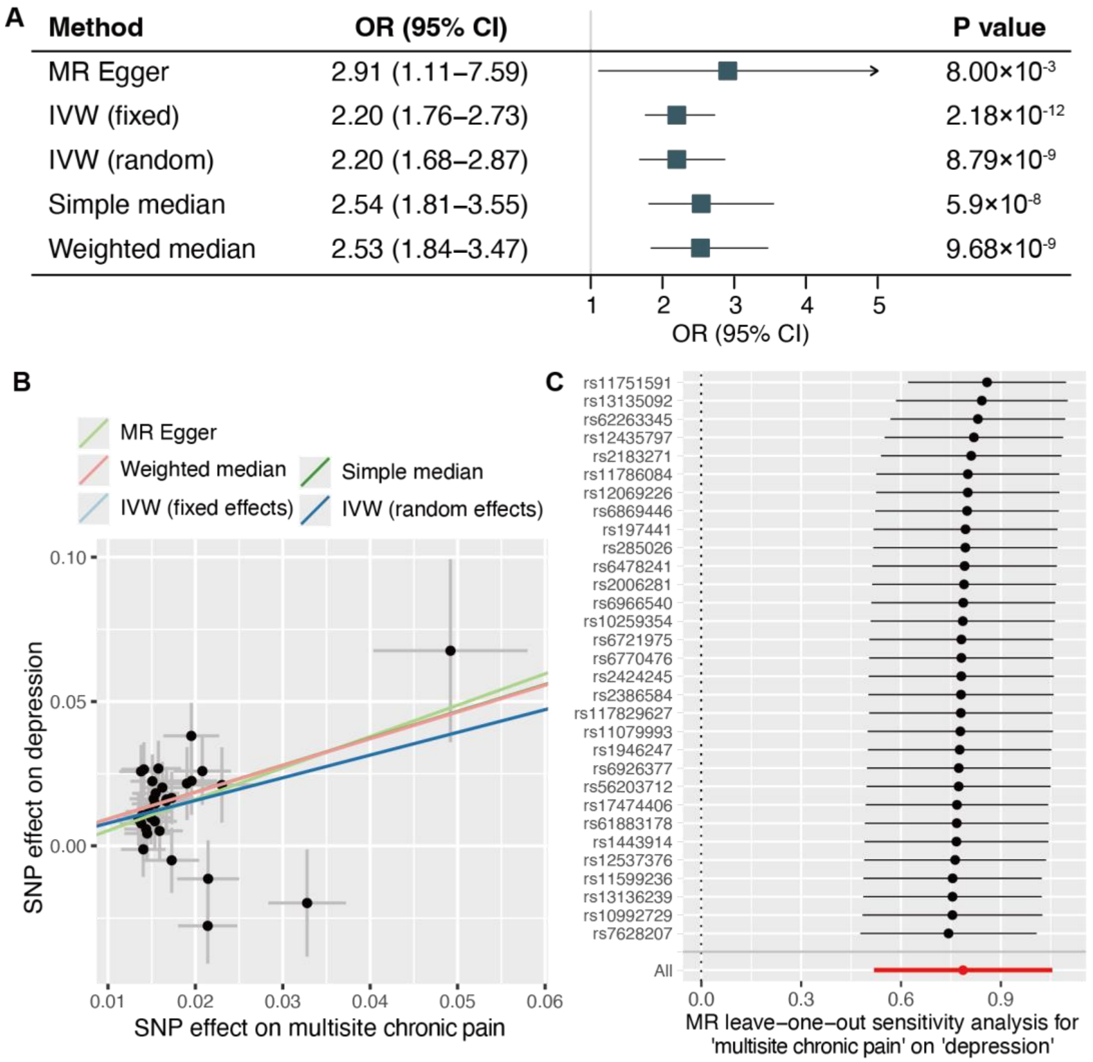

Fig. S10. Mendelian randomization plots for multisite chronic pain on risk of depression with depression SNPs selected from Psychiatric Genomics Consortium (PGC). (**A**) The causal effects of genetically predicted MCP on depression based on Mendelian randomization analyses. Dots: mean ORs; Horizontal lines: 95% CI. (**B**) Scatterplot of SNP effects on multisite chronic pain versus their effects on depression, with the slope of each line representing estimated MR effect per method. (**C**) Forest plot of leave-one-SNP-out sensitivity analysis. Dots: estimated beta coefficients; Horizontal lines: 95% CI. All P values were two-sided, and no adjustments were made for multiple comparisons.

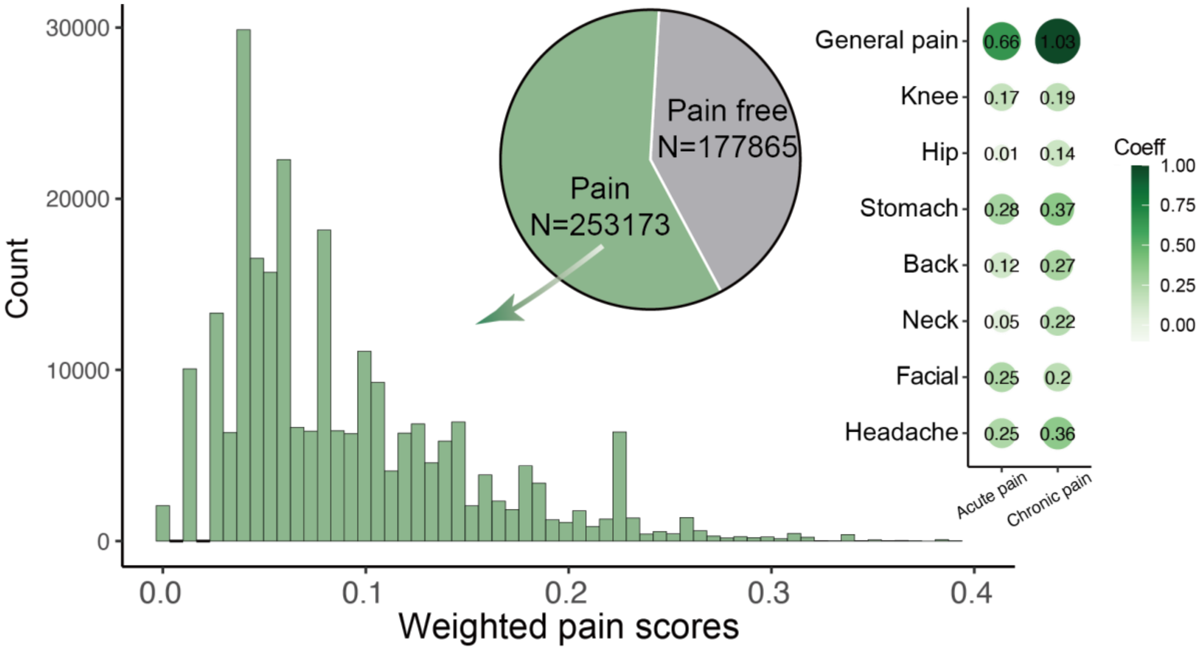

Fig. S11. Distribution of the composite pain scores and the derived beta weights for each pain condition. A higher score indicates more exposure to pain risks, and PF individuals will have a score of 0. The beta weights reflect the relative importance of each pain condition in calculating the composite pain scores.

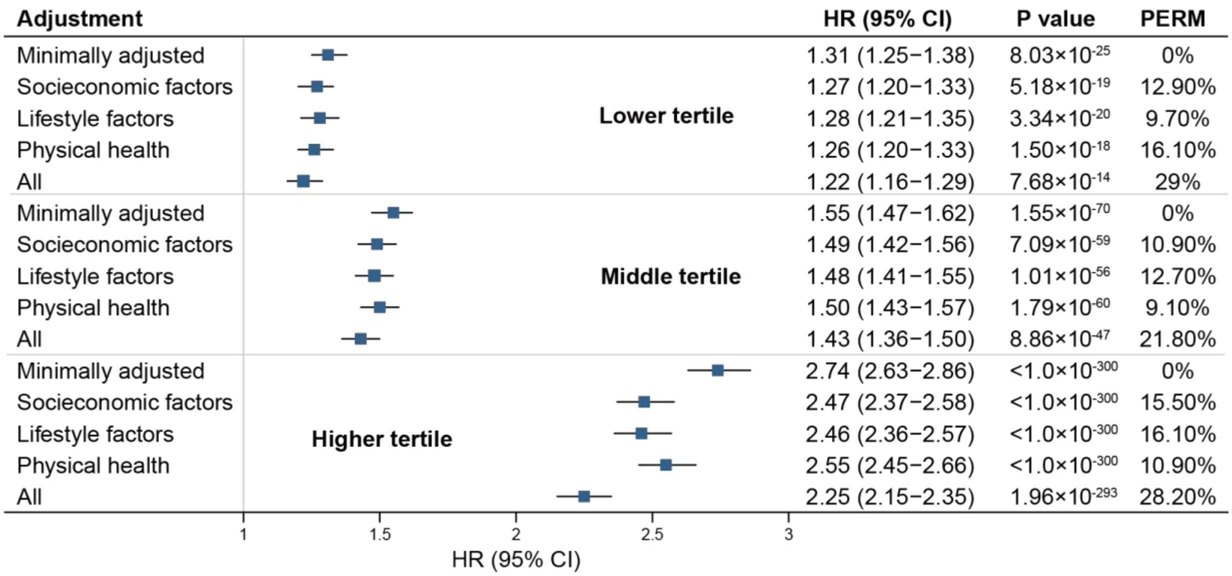

Fig. S12. Associations of the composite pain scores with depression incidence and the influence of covariates. In examining the prospective association between the tertile of the composite scores and depression incidence, we ran five models. Model 1 (minimally adjusted) included age, sex and race. On the basis of model 1, model 2 additionally included socioeconomic factors (material deprivation, family income, and education attainment); model 3 included lifestyle factors (smoking status, alcohol intake frequency, and sedentary status); model 4 included physical health factors (weight status, history of vascular or heart problems, history of cancer, and history of diabetes); model 5 included all 13 covariates.

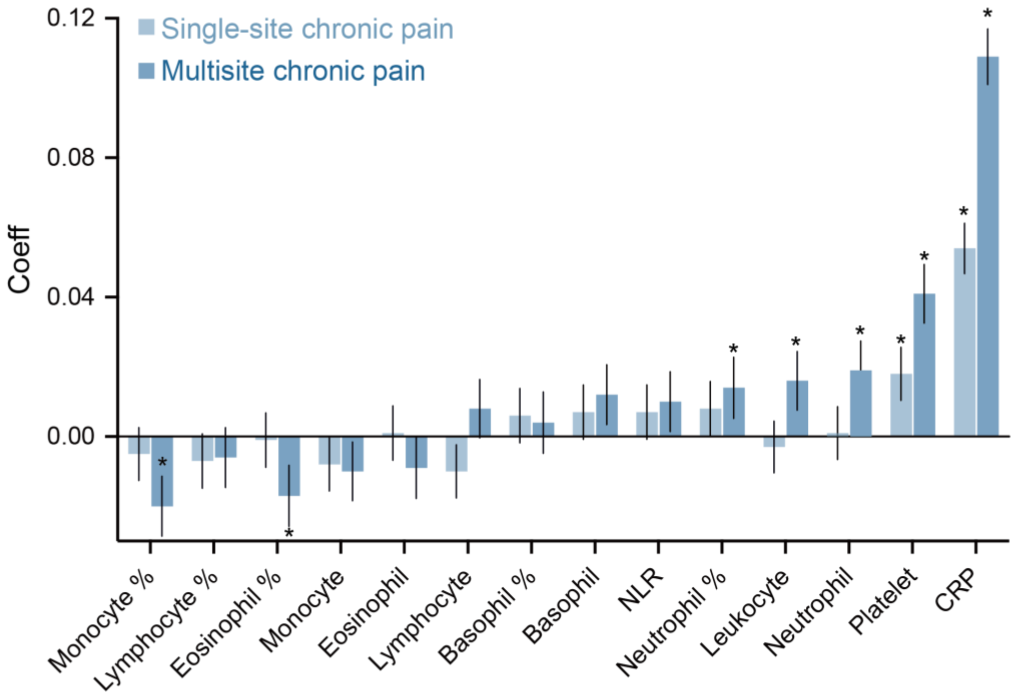

Fig. S13. Associations of SCP and MCP with inflammatory markers.

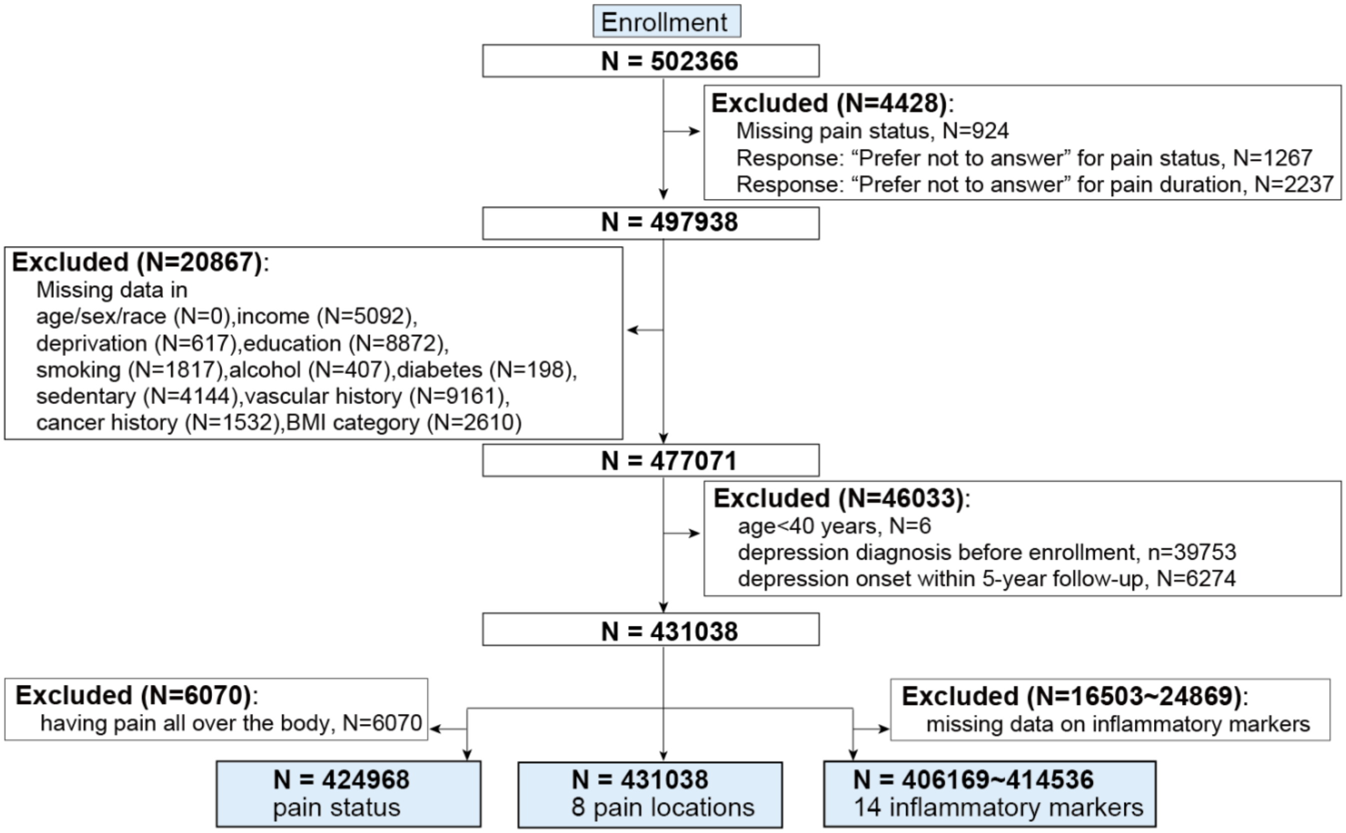

Fig. S14. Flowchart illustrating criteria for sample selection for all analyses performed in the present study.

Table S1. Baseline characteristics of participants with and without pain

	Whole population	Participants without Pain	Participants with acute/chronic pain at any site
Total N	431038	177865	253173
Age at baseline, years (mean, std)	56.6 (8.09)	56.8 (8.03)	56.46 (8.13)
Sex			
Females	229355 (53.21%)	92213 (51.84%)	137142 (54.17%)
Males	201683 (46.79%)	85652 (48.16%)	116031 (45.83%)
Race			
Ethnic minorities	23278 (5.4%)	7433 (4.18%)	15845 (6.26%)
White	407760 (94.6%)	170432 (95.82%)	237328 (93.74%)
Deprivation			
Lower tertile	149413 (34.66%)	65467 (36.81%)	83946 (33.16%)
Middle tertile	144488 (33.52%)	60497 (34.01%)	83991 (33.18%)
Higher tertile	137137 (31.82%)	51901 (29.18%)	85236 (33.67%)
Household Income			
Unknown	57164 (13.26%)	21796 (12.25%)	35368 (13.97%)
<£18000	78829 (18.29%)	27657 (15.55%)	51172 (20.21%)
£18000-£30999	94902 (22.02%)	39158 (22.02%)	55744 (22.02%)
£31000-£51999	99347 (23.05%)	42812 (24.07%)	56535 (22.33%)
£52000-£100000	79282 (18.39%)	36003 (20.24%)	43279 (17.09%)
>£100000	21514 (4.99%)	10439 (5.87%)	11075 (4.37%)
Educational attainment			
Less than college	287050 (66.6%)	111421 (62.64%)	175629 (69.37%)
Above College	143988 (33.4%)	66444 (37.36%)	77544 (30.63%)
Smoking status			
Never	239332 (55.52%)	103383 (58.12%)	135949 (53.7%)
Previous	149688 (34.73%)	59505 (33.46%)	90183 (35.62%)
Current	42018 (9.75%)	14977 (8.42%)	27041 (10.68%)
Alcohol intake frequency			
Daily or almost daily	89715 (20.81%)	40282 (22.65%)	49433 (19.53%)
3-4 times/week	102748 (23.84%)	45595 (25.63%)	57153 (22.57%)
1-2 times/week	112627 (26.13%)	46559 (26.18%)	66068 (26.1%)
1-3 times/month	47590 (11.04%)	18313 (10.3%)	29277 (11.56%)
Special occasions only	46921 (10.89%)	16441 (9.24%)	30480 (12.04%)
Never	31437 (7.29%)	10675 (6%)	20762 (8.2%)
Sedentary behavior			
0-4 hours/day	308865 (71.66%)	134160 (75.43%)	174705 (69.01%)
>4 hours/day	122173 (28.34%)	43705 (24.57%)	78468 (30.99%)

Vascular disease history			
No	305937 (70.98%)	131778 (74.09%)	174159 (68.79%)
Yes	125101 (29.02%)	46087 (25.91%)	79014 (31.21%)
Cancer history			
No	398218 (92.39%)	164556 (92.52%)	233662 (92.29%)
Yes	32820 (7.61%)	13309 (7.48%)	19511 (7.71%)
BMI category			
Normal weight	145213 (33.69%)	67917 (38.18%)	77296 (30.53%)
Overweight	184635 (42.83%)	76936 (43.26%)	107699 (42.54%)
Obese	101190 (23.48%)	33012 (18.56%)	68178 (26.93%)
Diabetes			
No	366705 (85.07%)	152433 (85.7%)	214272 (84.63%)
Yes	64333 (14.93%)	25432 (14.3%)	38901 (15.37%)

Table S2. Baseline characteristics by pain status

	Whole population	Pain free	Acute pain	Single-site chronic pain	Multisite chronic pain
Total N	424968	177865	70964	99383	76756
Age at baseline, years (mean, std)	56.59 (8.1)	56.8 (8.03)	55.28 (8.31)	56.73 (8.08)	57.16 (7.95)
Sex					
Females	225702 (53.11%)	92213 (51.84%)	35114 (49.48%)	52858 (53.19%)	45517 (59.3%)
Males	199266 (46.89%)	85652 (48.16%)	35850 (50.52%)	46525 (46.81%)	31239 (40.7%)
Race					
Ethnic minorities	22426 (5.28%)	7433 (4.18%)	4897 (6.9%)	5299 (5.33%)	4797 (6.25%)
White	402542 (94.72%)	170432 (95.82%)	66067 (93.1%)	94084 (94.67%)	71959 (93.75%)
Deprivation					
Lower	148004 (34.83%)	65467 (36.81%)	24506 (34.53%)	34137 (34.35%)	23894 (31.13%)
Middle	142749 (33.59%)	60497 (34.01%)	23684 (33.37%)	33758 (33.97%)	24810 (32.32%)
Higher	134215 (31.58%)	51901 (29.18%)	22774 (32.09%)	31488 (31.68%)	28052 (36.55%)
Income					
Unknown	55973 (13.17%)	21796 (12.25%)	9088 (12.81%)	13484 (13.57%)	11605 (15.12%)
<£18000	76709 (18.05%)	27657 (15.55%)	11459 (16.15%)	18372 (18.49%)	19221 (25.04%)
£18000-£30999	93645 (22.04%)	39158 (22.02%)	15102 (21.28%)	22253 (22.39%)	17132 (22.32%)
£31000-£51999	98400 (23.15%)	42812 (24.07%)	17328 (24.42%)	22792 (22.93%)	15468 (20.15%)
£52000-£100000	78806 (18.54%)	36003 (20.24%)	14133 (19.92%)	17774 (17.88%)	10896 (14.2%)
>£100000	21435 (5.04%)	10439 (5.87%)	3854 (5.43%)	4708 (4.74%)	2434 (3.17%)
Educational attainment					
Less than college	282165 (66.4%)	111421 (62.64%)	46009 (64.83%)	68014 (68.44%)	56721 (73.9%)
Above College	142803 (33.6%)	66444 (37.36%)	24955 (35.17%)	31369 (31.56%)	20035 (26.1%)
Smoking status					
Never	236254 (55.59%)	103383 (58.12%)	40472 (57.03%)	53670 (54%)	38729 (50.46%)
Previous	147656 (34.75%)	59505 (33.46%)	23559 (33.2%)	35676 (35.9%)	28916 (37.67%)
Current	41058 (9.66%)	14977 (8.42%)	6933 (9.77%)	10037 (10.1%)	9111 (11.87%)
Alcohol intake frequency					
Daily or almost daily	88952 (20.93%)	40282 (22.65%)	14282 (20.13%)	20611 (20.74%)	13777 (17.95%)
3-4 times/week	101894 (23.98%)	45595 (25.63%)	17381 (24.49%)	23524 (23.67%)	15394 (20.06%)
1-2 times/week	111267 (26.18%)	46559 (26.18%)	19289 (27.18%)	26262 (26.43%)	19157 (24.96%)
1-3 times/month	46851 (11.02%)	18313 (10.3%)	8015 (11.29%)	11003 (11.07%)	9520 (12.4%)
Special occasions only	45730 (10.76%)	16441 (9.24%)	7373 (10.39%)	10766 (10.83%)	11150 (14.53%)
Never	30274 (7.12%)	10675 (6%)	4624 (6.52%)	7217 (7.26%)	7758 (10.11%)
Sedentary behavior					
0-4 hours/day	305417 (71.87%)	134160 (75.43%)	52117 (73.44%)	70073 (70.51%)	49067 (63.93%)
>4 hours/day	119551 (28.13%)	43705 (24.57%)	18847 (26.56%)	29310 (29.49%)	27689 (36.07%)

Vascular history					
No	302584 (71.2%)	131778 (74.09%)	51909 (73.15%)	69620 (70.05%)	49277 (64.2%)
Yes	122384 (28.8%)	46087 (25.91%)	19055 (26.85%)	29763 (29.95%)	27479 (35.8%)
Cancer history					
No	392759 (92.42%)	164556 (92.52%)	66138 (93.2%)	91853 (92.42%)	70212 (91.47%)
Yes	32209 (7.58%)	13309 (7.48%)	4826 (6.8%)	7530 (7.58%)	6544 (8.53%)
BMI category					
Normal weight	143934 (33.87%)	67917 (38.18%)	24218 (34.13%)	31265 (31.46%)	20534 (26.75%)
Overweight	182243 (42.88%)	76936 (43.26%)	30877 (43.51%)	42569 (42.83%)	31861 (41.51%)
Obese	98791 (23.25%)	33012 (18.56%)	15869 (22.36%)	25549 (25.71%)	24361 (31.74%)
Diabetes					
No	361998 (85.18%)	152433 (85.7%)	60941 (85.88%)	84593 (85.12%)	64031 (83.42%)
Yes	62970 (14.82%)	25432 (14.3%)	10023 (14.12%)	14790 (14.88%)	12725 (16.58%)

Table S3. Associations of SCP, MCP, and covariates with the risk of depression

	HR (95% CI)	P value	Participants	Events
Pain status				
Pain free	1.00 (Ref)		177865	3822
Acute pain	1.27 (1.2-1.34)	1.35E-17	70964	1978
Single site chronic pain	1.44 (1.38-1.51)	4.18E-53	99383	3343
Multisite chronic pain	2.12 (2.03-2.22)	4.13E-239	76756	4313
Age				
Middle age	1.00 (Ref)		342841	10551
Older	0.93 (0.89-0.97)	0.00148	82127	2905
Sex				
Female	1.00 (Ref)		225702	8210
Male	0.74 (0.71-0.76)	3.00E-61	199266	5246
Race				
Ethnic minorities	1.00 (Ref)		22426	595
White	1.46 (1.34-1.59)	3.41E-18	402542	12861
Deprivation				
Low	1.00 (Ref)		148004	3861
Middle	1.06 (1.02-1.11)	0.00675	142749	4272
High	1.22 (1.16-1.27)	1.18E-18	134215	5323
Income				
Unknown	1.00 (Ref)		55973	2048
<£18000	1.2 (1.14-1.27)	2.65E-11	76709	3799
£18000-£30999	0.98 (0.93-1.04)	0.472	93645	3170
£31000-£51999	0.84 (0.79-0.89)	6.24E-09	98400	2599
£52000-£100000	0.70 (0.65-0.75)	1.57E-24	78806	1574
>£100000	0.47 (0.41-0.53)	4.50E-30	21435	266
Educational attainment				
Less than college	1.00 (Ref)		282165	10090
university_education1	0.92 (0.89-0.96)	0.000234	142803	3366
Smoking status				
Never	1.00 (Ref)		236254	6260
Ever	1.23 (1.19-1.28)	6.14E-27	147656	5091
Current	1.73 (1.64-1.82)	3.95E-97	41058	2105
Alcohol intake frequency				
Daily or almost daily	1.00 (Ref)		88952	2496
3-4 times/week	0.92 (0.87-0.97)	0.0018	101894	2570
1-2 times/week	0.99 (0.93-1.04)	0.579	111267	3327
1-3 times/month	1.08 (1.02-1.15)	0.0133	46851	1675

Special occasions only	1.16 (1.09-1.23)	3.79E-06	45730	1969
Never	1.32 (1.24-1.42)	8.12E-16	30274	1419
Sedentary behavior				
0-4 hours/day	1.00 (Ref)		305417	8602
>4 hours/day	1.07 (1.03-1.11)	0.000602	119551	4854
Vascular history				
No	1.00 (Ref)		302584	8475
Yes	1.23 (1.18-1.28)	4.88E-27	122384	4981
Cancer history				
No	1.00 (Ref)		392759	12236
Yes	1.09 (1.03-1.16)	0.00345	32209	1220
BMI category				
Normal weight	1.00 (Ref)		143934	3815
Overweight	1.09 (1.04-1.13)	0.000156	182243	5463
Obese	1.26 (1.2-1.32)	3.34E-22	98791	4178
Diabetes				
No	1.00 (Ref)		361998	11100
Yes	1.05 (1.01-1.1)	0.0236	62970	2356

Table S4. Associations between pain status and incident depression by age group and education level

	HR (95% CI)	P	HR (95% CI)	P	P interaction
	Age≤65 years		Age>65 years		
Acute pain	1.29 (1.21-1.37)	9.38E-16	1.23 (1.09-1.38)	7.58E-04	0.49
SCP	1.51 (1.43-1.59)	5.44E-52	1.26 (1.14-1.39)	3.60E-06	1.53E-03
MCP	2.26 (2.14-2.37)	7.00E-213	1.72 (1.57-1.89)	4.70E-30	4.40E-07
	Less than college		Above College		
Acute pain	1.34 (1.25-1.42)	3.61E-19	1.12 (1.00-1.24)	0.0434	3.87E-03
SCP	1.43 (1.35-1.51)	9.14E-37	1.51 (1.38-1.65)	9.73E-20	0.31
MCP	2.13 (2.02-2.24)	2.39E-177	2.11 (1.93-2.31)	6.36E-61	0.88

Table S5. Baseline characteristics of participants with acute pain, SCP, and MCP and matched pain-free participants

	Acute pain			Single-site chronic pain			Multisite chronic pain		
	Matched PF participants	Acute pain participants	P	Matched PF participants	SCP participants	P	Matched PF participants	MCP participants	P
Original participants	177865	70964		177865	99383		177865	76756	
Matched participants	62626	62626		89040	89040		62805	62805	
Age			1			1			1
Mid-age	53657 (85.68%)	53657 (85.68%)		72862 (81.83%)	72862 (81.83%)		51212 (81.54%)	51212 (81.54%)	
Older age	8969 (14.32%)	8969 (14.32%)		16178 (18.17%)	16178 (18.17%)		11593 (18.46%)	11593 (18.46%)	
Sex			1			0.476			0.154
Females	31100 (49.66%)	31100 (49.66%)		47394 (53.23%)	47243 (53.06%)		36667 (58.38%)	36417 (57.98%)	
Males	31526 (50.34%)	31526 (50.34%)		41646 (46.77%)	41797 (46.94%)		26138 (41.62%)	26388 (42.02%)	
Race			0.816			1			1
Ethnic minorities	2459 (3.93%)	2476 (3.95%)		2886 (3.24%)	2886 (3.24%)		2201 (3.5%)	2201 (3.5%)	
White	60167 (96.07%)	60150 (96.05%)		86154 (96.76%)	86154 (96.76%)		60604 (96.5%)	60604 (96.5%)	
Deprivation			0.594			0.452			0.669
Lower	22210 (35.46%)	22350 (35.69%)		31831 (35.75%)	31688 (35.59%)		20971 (33.39%)	21106 (33.61%)	
Middle	21021 (33.57%)	21033 (33.59%)		30537 (34.3%)	30437 (34.18%)		20925 (33.32%)	20801 (33.12%)	
Higher	19395 (30.97%)	19243 (30.73%)		26672 (29.96%)	26915 (30.23%)		20909 (33.29%)	20898 (33.27%)	
Income			0.868			0.137			0.318
Unknown	7430 (11.86%)	7533 (12.03%)		11230 (12.61%)	11146 (12.52%)		8566 (13.64%)	8654 (13.78%)	
<£18000	9517 (15.2%)	9544 (15.24%)		15235 (17.11%)	15342 (17.23%)		13621 (21.69%)	13495 (21.49%)	
£18000-£30999	13500 (21.56%)	13373 (21.35%)		20080 (22.55%)	20358 (22.86%)		14631 (23.3%)	14515 (23.11%)	
£31000-£51999	15838 (25.29%)	15752 (25.15%)		21431 (24.07%)	20990 (23.57%)		13930 (22.18%)	13832 (22.02%)	
£52000-£100000	12962 (20.7%)	13008 (20.77%)		16884 (18.96%)	16926 (19.01%)		9957 (15.85%)	10093 (16.07%)	
>£100000	3379 (5.4%)	3416 (5.45%)		4180 (4.69%)	4278 (4.8%)		2100 (3.34%)	2216 (3.53%)	
Education			1			1			1
Less than college	40922 (65.34%)	40922 (65.34%)		60781 (68.26%)	60781 (68.26%)		46024 (73.28%)	46024 (73.28%)	
Above College	21704 (34.66%)	21704 (34.66%)		28259 (31.74%)	28259 (31.74%)		16781 (26.72%)	16781 (26.72%)	
Smoking status			1			1			1
Never	36811 (58.78%)	36811 (58.78%)		49698 (55.82%)	49698 (55.82%)		33622 (53.53%)	33622 (53.53%)	
Previous	20696 (33.05%)	20696 (33.05%)		31739 (35.65%)	31739 (35.65%)		23305 (37.11%)	23305 (37.11%)	
Current	5119 (8.17%)	5119 (8.17%)		7603 (8.54%)	7603 (8.54%)		5878 (9.36%)	5878 (9.36%)	
Alcohol intake			0.923			0.14			0.605
Daily/almost daily	12923 (20.64%)	12922 (20.63%)		19037 (21.38%)	18914 (21.24%)		12197 (19.42%)	12139 (19.33%)	
3-4 times/week	15998 (25.55%)	15997 (25.54%)		22057 (24.77%)	22049 (24.76%)		13928 (22.18%)	13817 (22%)	
1-2 times/week	17605 (28.11%)	17603 (28.11%)		24273 (27.26%)	23956 (26.9%)		16777 (26.71%)	16648 (26.51%)	
1-3 times/month	6752 (10.78%)	6706 (10.71%)		9569 (10.75%)	9787 (10.99%)		7499 (11.94%)	7648 (12.18%)	

Special occasions only	5870 (9.37%)	5831 (9.31%)		8782 (9.86%)	8824 (9.91%)		7816 (12.44%)	7877 (12.54%)	
Never	3478 (5.55%)	3567 (5.7%)		5322 (5.98%)	5510 (6.19%)		4588 (7.31%)	4676 (7.45%)	
Sedentary behavior			0.361			0.848			0.171
0-4 hours/day	47406 (75.7%)	47266 (75.47%)		64980 (72.98%)	64943 (72.94%)		43014 (68.49%)	42787 (68.13%)	
>4 hours/day	15220 (24.3%)	15360 (24.53%)		24060 (27.02%)	24097 (27.06%)		19791 (31.51%)	20018 (31.87%)	
Vascular history			1			0.153			1
No	47482 (75.82%)	47482 (75.82%)		64846 (72.83%)	64576 (72.52%)		43573 (69.38%)	43573 (69.38%)	
Yes	15144 (24.18%)	15144 (24.18%)		24194 (27.17%)	24464 (27.48%)		19232 (30.62%)	19232 (30.62%)	
Cancer history			1			1			1
No	59831 (95.54%)	59831 (95.54%)		84166 (94.53%)	84166 (94.53%)		59401 (94.58%)	59401 (94.58%)	
Yes	2795 (4.46%)	2795 (4.46%)		4874 (5.47%)	4874 (5.47%)		3404 (5.42%)	3404 (5.42%)	
BMI category						0.454			0.966
Normal weight	21906 (34.98%)	22049 (35.21%)		29255 (32.86%)	29503 (33.13%)		18497 (29.45%)	18528 (29.5%)	
Overweight	28039 (44.77%)	27857 (44.48%)		39437 (44.29%)	39296 (44.13%)		27724 (44.14%)	27679 (44.07%)	
Obese	12681 (20.25%)	12720 (20.31%)		20348 (22.85%)	20241 (22.73%)		16584 (26.41%)	16598 (26.43%)	
Diabetes			1			1			1
No	55781 (89.07%)	55781 (89.07%)		78106 (87.72%)	78106 (87.72%)		54972 (87.53%)	54972 (87.53%)	
Yes	6845 (10.93%)	6845 (10.93%)		10934 (12.28%)	10934 (12.28%)		7833 (12.47%)	7833 (12.47%)	

Table S6. Causal relationships between pain conditions and depression based on Mendelian randomization analyses using summary statistics for depression from iPSYCH

	P value	Odds Ratio, 95% CI			Heterogeneity		Pleiotropy		Number of SNPs
		OR	Lower	Upper	Q statistics	P value	Intercept	P value	
**Headache**									
MR Egger	0.36	0.93	0.57	1.51					
IVW (fixed)	1.49E-05	0.68	0.57	0.81					
IVW (random)	0.012	0.68	0.5	0.92	65.52	1.80E-06	-0.00039	0.945	23
Simple median	0.044	0.73	0.54	0.99					
Weighted median	0.069	0.77	0.58	1.02					
**Facial pain**									
MR Egger	0.296	2.21	0.1	49.76					
IVW (fixed)	0.115	2.31	0.81	6.53					
IVW (random)	0.220	2.31	0.61	8.77	21.13	0.0485	-0.00268	0.691	14
Simple median	0.222	2.55	0.57	11.44					
Weighted median	0.282	2.31	0.5	10.62					
**Neck pain**									
MR Egger	0.074	0.02	0	803.83					
IVW (fixed)	2.49E-05	0.29	0.16	0.51					
IVW (random)	0.109	0.29	0.06	1.32	4.49	0.0342	-0.11	0.385	3
Simple median	0.004	0.25	0.1	0.64					
Weighted median	0.015	0.31	0.12	0.8					
**Back pain**									
MR Egger	0.307	1.19	0.61	2.32					
IVW (fixed)	0.185	0.82	0.61	1.1					
IVW (random)	0.213	0.82	0.6	1.12	19.77	0.346	0.005	0.211	20
Simple median	0.802	0.95	0.62	1.45					
Weighted median	0.729	0.93	0.6	1.43					
**Stomach pain**									
MR Egger	0.066	9.08	0.35	234.39					
IVW (fixed)	0.450	0.71	0.3	1.71	2.79	0.732	0.0168	0.0667	7
IVW (random)	0.519	0.71	0.26	1.99					

Simple median	0.384	0.56	0.15	2.08					
Weighted median	0.367	0.55	0.15	2.01					
**Hip pain**									
MR Egger	0.277	1.87	0.23	15.26					
IVW (fixed)	0.380	0.67	0.28	1.63					
IVW (random)	0.337	0.67	0.3	1.51	3.11	0.54	0.0089	0.36	6
Simple median	0.344	0.58	0.19	1.79					
Weighted median	0.398	0.6	0.18	1.96					
**Knee pain**									
MR Egger	0.119	0.6	0.26	1.4					
IVW (fixed)	0.005	0.59	0.41	0.86					
IVW (random)	0.016	0.59	0.39	0.91	20.26	0.122	-0.00047	0.914	16
Simple median	0.468	0.82	0.48	1.39					
Weighted median	0.303	0.75	0.44	1.29					
**General pain**									
MR Egger	0.147	0.47	0.1	2.16					
IVW (fixed)	0.525	0.86	0.55	1.36					
IVW (random)	0.763	0.86	0.33	2.24	48.03	3.09E-06	-0.00794	0.141	14
Simple median	0.364	0.64	0.25	1.67					
Weighted median	0.615	1.16	0.65	2.07					
**Multiset chronic pain**									
MR Egger	0.008	2.29	1.1	4.77					
IVW (fixed)	1.06E-37	2.07	1.85	2.31					
IVW (random)	1.06E-09	2.07	1.64	2.62	106.02	5.95E-12	0.0127	0.156	27
Simple median	1.68E-11	2.04	1.66	2.51					
Weighted median	6.52E-11	1.99	1.62	2.45					

Table S7. Characteristics of 27 SNPs associated with multiple chronic pain

SNP	Effect allele	Other allele	EAF	Beta	SE	P value
rs7628207	T	C	0.18	0.0195	0.0032	8.40E-10
rs62263345	A	G	0.86	-0.0215	0.0035	1.40E-09
rs6770476	C	T	0.71	-0.0154	0.0027	9.40E-09
rs56203712	A	G	0.76	0.0196	0.0029	2.30E-11
rs13135092	A	G	0.92	-0.0328	0.0044	1.50E-13
rs13136239	G	A	0.66	0.0141	0.0026	3.60E-08
rs6869446	T	C	0.62	-0.0144	0.0025	9.50E-09
rs17474406	G	A	0.98	-0.0492	0.0088	2.40E-08
rs1946247	T	G	0.14	-0.019	0.0035	4.90E-08
rs11751591	G	A	0.85	0.0214	0.0034	2.70E-10
rs6926377	A	C	0.71	-0.0155	0.0027	7.90E-09
rs10259354	G	A	0.31	0.0147	0.0026	3.00E-08
rs6966540	T	C	0.63	-0.0139	0.0025	3.30E-08
rs12537376	A	G	0.61	0.0151	0.0025	1.70E-09
rs11786084	G	A	0.67	-0.0145	0.0026	2.30E-08
rs10992729	C	T	0.67	0.0158	0.0026	1.10E-09
rs6478241	A	G	0.36	0.0149	0.0025	3.10E-09
rs2183271	T	C	0.64	-0.014	0.0025	3.10E-08
rs11599236	T	C	0.59	0.0138	0.0025	3.30E-08
rs61883178	C	A	0.83	-0.0208	0.0033	2.00E-10
rs1443914	T	C	0.47	0.0162	0.0024	2.80E-11
rs12435797	G	T	0.82	-0.0173	0.0031	3.70E-08
rs2006281	C	T	0.5	0.0135	0.0024	3.40E-08
rs2386584	T	G	0.61	-0.0166	0.0025	2.80E-11
rs285026	G	T	0.43	-0.0138	0.0025	1.90E-08
rs11079993	G	T	0.62	-0.0173	0.0025	5.70E-12
rs2424245	C	T	0.87	0.0230	0.0037	3.70E-10

Valid instrumental variables (IVs) had to satisfy the following three assumptions: (1) association with the risk exposure of interest (relevance); (2) no common cause with the outcome (independence); (3) affect the outcome only through the risk exposure (exclusion restriction).

Table S8. Causal relationships between pain conditions and depression based on Mendelian randomization analyses using summary statistics for depression from Psychiatric Genomics Consortium

	P value	Odds Ratio, 95% CI			Heterogeneity		Pleiotropy		Number of SNPs
		OR	Lower	Upper	Q statistics	P value	Intercept	P value	
**Headache**									
MR Egger	0.062	0.45	0.17	1.22					
IVW (fixed)	0.024	0.64	0.44	0.94					
IVW (random)	0.021	0.64	0.44	0.93	19.45	0.493	-0.00458	0.498	22
Simple median	0.358	0.77	0.44	1.35					
Weighted median	0.059	0.59	0.34	1.02					
**Facial pain**									
MR Egger	0.445	0.73	0	136.42					
IVW (fixed)	0.213	3.66	0.48	28.09					
IVW (random)	0.222	3.66	0.46	29.24	15.42	0.35	-0.00381	0.696	16
Simple median	0.405	3.17	0.21	47.85					
Weighted median	0.238	5.52	0.32	93.92					
**Neck pain**									
MR Egger	0.189	0.04	0	5559.15					
IVW (fixed)	0.038	0.27	0.08	0.93					
IVW (random)	0.033	0.27	0.08	0.9	0.40	0.525	-0.0925	0.437	3
Simple median	0.108	0.28	0.06	1.32					
Weighted median	0.106	0.28	0.06	1.31					
**Back pain**									
MR Egger	0.326	1.42	0.3	6.64					
IVW (fixed)	0.228	0.68	0.37	1.27					
IVW (random)	0.189	0.68	0.39	1.21	14.29	0.71	0.0102	0.203	20
Simple median	0.098	0.49	0.21	1.14					
Weighted median	0.091	0.49	0.21	1.12					
**Stomach pain**									
MR Egger	0.348	2.57	0.02	402.76					
IVW (fixed)	0.748	1.35	0.22	8.45	1.05	0.958	0.00596	0.635	7
IVW (random)	0.491	1.35	0.57	3.18					

Simple median	0.823	1.32	0.12	15.02					
Weighted median	0.854	1.24	0.13	11.73					
**Hip pain**									
MR Egger	0.277	0.46	0.03	6.81					
IVW (fixed)	0.167	0.44	0.14	1.41					
IVW (random)	0.171	0.44	0.14	1.43	7.58	0.371	0.00675	0.499	9
Simple median	0.732	0.73	0.12	4.41					
Weighted median	0.332	0.45	0.09	2.27					
**Knee pain**									
MR Egger	0.409	1.15	0.28	4.7					
IVW (fixed)	0.655	0.84	0.38	1.83					
IVW (random)	0.669	0.84	0.37	1.89	14.54	0.485	0.0122	0.106	17
Simple median	0.969	0.98	0.3	3.18					
Weighted median	0.928	0.95	0.31	2.91					
**General pain**									
MR Egger	0.209	0.54	0.12	2.53					
IVW (fixed)	0.376	0.67	0.27	1.64					
IVW (random)	0.342	0.67	0.29	1.54	7.58	0.75	-0.00863	0.122	13
Simple median	0.195	0.29	0.04	1.9					
Weighted median	0.783	0.85	0.28	2.61					
**Multiset chronic pain**									
MR Egger	0.008	2.91	1.11	7.59					
IVW (fixed)	2.18E-12	2.2	1.76	2.73					
IVW (random)	8.79E-09	2.2	1.68	2.87	42.71	0.0484	0.0127	0.253	31
Simple median	5.90E-08	2.54	1.81	3.55					
Weighted median	9.68E-09	2.53	1.84	3.47					

Table S9. Associations between tertiles of the composite pain scores and incident depression

	HR (95% CI)	P value	Participants	Events
Pain free	Ref	Ref	177865	3822
Lower tertile	1.22 (1.16-1.29)	7.68E-14	80841	2260
Middle tertile	1.43 (1.36-1.50)	8.86E-47	88045	2916
Higher tertile	2.25 (2.15-2.35)	1.96E-293	84287	4949

Table S10. Associations between inflammatory markers and the composite pain scores

	Coeff	N	T value	P value	Cohen's d	95% CI	
						Lower	Upper
CRP	0.05	406169	34.37	1.51E-258	0.1	0.094	0.106
Basophil count	0.007	413797	4.51	6.57E-06	0.014	0.008	0.020
Basophil %	0.003	413801	1.62	0.104	0.006	-0.0003	0.012
Eosinophil count	-0.002	413797	-1.25	0.212	-0.004	-0.010	0.002
Eosinophil %	-0.006	413801	-4.07	4.69E-05	-0.012	-0.018	-0.0057
Leukocyte count	0.013	414533	8.26	1.50E-16	0.026	0.020	0.032
Lymphocyte count	0.003	413790	1.83	0.0673	0.006	0.0001	0.012
Lymphocyte %	-0.007	413801	-4.59	4.34E-06	-0.014	-0.020	-0.008
Monocyte count	-0.003	413797	-1.82	0.0688	-0.006	-0.012	-0.0001
Monocyte %	-0.011	413801	-6.97	3.17E-12	-0.022	-0.028	-0.016
Neutrophil count	0.014	413792	9.26	2.11E-20	0.028	0.022	0.034
Neutrophil %	0.01	413801	6.33	2.50E-10	0.02	0.014	0.026
NLR	0.009	413788	5.97	2.39E-09	0.018	0.0117	0.024
Platelet count	0.025	414535	16.46	6.99E-61	0.05	0.044	0.056

Table S11. Linear and non-linear associations between inflammatory markers and the risk of depression incidence

	Non-linear model		Linear model	
	$P_{overall}$	$P_{nonlinear}$	HR (95% CI)	P_{linear}
CRP	1.56E-08	0.170	1.06 (1.04-1.08)	6.42E-09
Basophil count	0.006	0.015	1.02 (1.00-1.04)	0.0147
Basophil_percentage	0.973	0.959	1.00 (0.99-1.02)	0.82
Eosinophil count	0.183	0.215	1.01 (0.99-1.03)	0.177
Eosinophil_percentage	0.298	0.205	0.99 (0.97-1.01)	0.37
Leukocyte count	3.33E-08	0.645	1.05 (1.03-1.07)	3.49E-09
Lymphocyte count	0.272	0.130	1.00 (0.99-1.02)	0.57
Lymphocyte_percentage	2.41E-06	0.392	0.96 (0.94-0.97)	7.06E-07
Monocyte count	0.072	0.518	1.02 (1.00-1.04)	0.0266
Monocyte_percentage	0.031	0.079	0.98 (0.97-1.00)	0.0547
Neutrophil count	3.13E-10	0.201	1.06 (1.04-1.08)	5.93E-11
Neutrophil_percentage	2.89E-06	0.056	1.04 (1.02-1.06)	3.12E-06
NLR	1.97E-06	0.518	1.05 (1.03-1.06)	2.79E-07
Platelet count	1.84E-07	0.006	1.04 (1.02-1.06)	1.48E-06

Table S12. UK Biobank showcase variables used in the study

Variable	Field ID	UK Biobank showcase link
**Demographic, socioeconomic, lifestyle, and health measures**		
Age	21003	https://biobank.ndph.ox.ac.uk/showcase/field.cgi?id=21003
Self-reported sex	31	https://biobank.ndph.ox.ac.uk/showcase/field.cgi?id=31
Assessment date	53	https://biobank.ndph.ox.ac.uk/showcase/field.cgi?id=53
Assessment center	54	https://biobank.ndph.ox.ac.uk/showcase/field.cgi?id=54
Race	21000	https://biobank.ndph.ox.ac.uk/showcase/field.cgi?id=21000
Material deprivation	22189	https://biobank.ndph.ox.ac.uk/showcase/field.cgi?id=22189
Educational attainment	6138	https://biobank.ndph.ox.ac.uk/showcase/field.cgi?id=6138
Family income	738	https://biobank.ndph.ox.ac.uk/showcase/field.cgi?id=738
Smoking status	20116	https://biobank.ndph.ox.ac.uk/showcase/field.cgi?id=20116
Alcohol intake	1558	https://biobank.ndph.ox.ac.uk/showcase/field.cgi?id=1558
Time spent watching TV	1070	https://biobank.ndph.ox.ac.uk/showcase/field.cgi?id=1070
Waist	48	https://biobank.ndph.ox.ac.uk/showcase/field.cgi?id=48
Body mass index	21001	https://biobank.ndph.ox.ac.uk/showcase/field.cgi?id=21001
Diabetes diagnosed by doctor	2443	https://biobank.ndph.ox.ac.uk/showcase/field.cgi?id=2443
Glucose	30740	https://biobank.ndph.ox.ac.uk/showcase/field.cgi?id=30740
Vascular/heart problems diagnosed by doctor	6150	https://biobank.ndph.ox.ac.uk/showcase/field.cgi?id=6150
Cancer diagnosed by doctor	2453	https://biobank.ndph.ox.ac.uk/showcase/field.cgi?id=2453
Medication for pain relief, constipation, heartburn	6154	https://biobank.ndph.ox.ac.uk/showcase/field.cgi?id=6154
**First occurrence of depression**		
Date F32 first reported (depressive episode)	130894	https://biobank.ndph.ox.ac.uk/showcase/field.cgi?id=130894
Source of report of F32 (depressive episode)	130895	https://biobank.ndph.ox.ac.uk/showcase/field.cgi?id=130895
Date F33 first reported (recurrent depressive disorder)	130896	https://biobank.ndph.ox.ac.uk/showcase/field.cgi?id=130896
Source of report of F33 (recurrent depressive disorder)	130897	https://biobank.ndph.ox.ac.uk/showcase/field.cgi?id=130897
Date F40 first reported (phobic anxiety disorders)	130904	https://biobank.ndph.ox.ac.uk/showcase/field.cgi?id=130904
Source of report of F40 (phobic anxiety disorders)	130905	https://biobank.ndph.ox.ac.uk/showcase/field.cgi?id=130905
Date F41 first reported (other anxiety disorders)	130906	https://biobank.ndph.ox.ac.uk/showcase/field.cgi?id=130906
Source of report of F41 (other anxiety disorders)	130907	https://biobank.ndph.ox.ac.uk/showcase/field.cgi?id=130907
Reason lost to follow-up	190	https://biobank.ndph.ox.ac.uk/showcase/field.cgi?id=190
Date lost to follow-up	191	https://biobank.ndph.ox.ac.uk/showcase/field.cgi?id=191
**Pain locations and duration**		

Pain type(s) experienced in last month	6159	https://biobank.ndph.ox.ac.uk/showcase/field.cgi?id=6159
General pain for 3+ months	2956	https://biobank.ndph.ox.ac.uk/showcase/field.cgi?id=2956
Neck/shoulder pain for 3+ months	3404	https://biobank.ndph.ox.ac.uk/showcase/field.cgi?id=3404
Hip pain for 3+ months	3414	https://biobank.ndph.ox.ac.uk/showcase/field.cgi?id=3414
Stomach/abdominal pain for 3+ months	3741	https://biobank.ndph.ox.ac.uk/showcase/field.cgi?id=3741
Back pain for 3+ months	3571	https://biobank.ndph.ox.ac.uk/showcase/field.cgi?id=3571
Knee pain for 3+ months	3773	https://biobank.ndph.ox.ac.uk/showcase/field.cgi?id=3773
Headaches for 3+ months	3799	https://biobank.ndph.ox.ac.uk/showcase/field.cgi?id=3799
Facial pains for 3+ months	4067	https://biobank.ndph.ox.ac.uk/showcase/field.cgi?id=4067
**Inflammatory markers**		
C-reactive protein	30710	https://biobank.ndph.ox.ac.uk/showcase/field.cgi?id=30710
Leukocyte	30000	https://biobank.ndph.ox.ac.uk/showcase/field.cgi?id=30000
Platelet count	30080	https://biobank.ndph.ox.ac.uk/showcase/field.cgi?id=30080
Lymphocyte count	30120	https://biobank.ndph.ox.ac.uk/showcase/field.cgi?id=30120
Monocyte count	30130	https://biobank.ndph.ox.ac.uk/showcase/field.cgi?id=30130
Neutrophil count	30140	https://biobank.ndph.ox.ac.uk/showcase/field.cgi?id=30140
Lymphocyte percentage	30180	https://biobank.ndph.ox.ac.uk/showcase/field.cgi?id=30180
Monocyte percentage	30190	https://biobank.ndph.ox.ac.uk/showcase/field.cgi?id=30190
Neutrophil percentage	30200	https://biobank.ndph.ox.ac.uk/showcase/field.cgi?id=30200
Basophil percentage	30220	https://biobank.ndph.ox.ac.uk/showcase/field.cgi?id=30220
Eosinophil percentage	30210	https://biobank.ndph.ox.ac.uk/showcase/field.cgi?id=30210
Basophil count	30160	https://biobank.ndph.ox.ac.uk/showcase/field.cgi?id=30160
Eosinophil count	30150	https://biobank.ndph.ox.ac.uk/showcase/field.cgi?id=30150

Table S13. Information and source of GWAS data used for Mendelian randomization analyses

Traits	Participants	Source/Doi
MDD	N=143,256*<br>European ancestry<br>(45,591 cases and 97,674 controls)	▪ https://figshare.com/articles/dataset/MDD2_MDD2018_GWAS_sumstats_w_o_UKBB/21655784;<br>▪ doi: 10.1038/s41588-018-0090-3
	N=674452**<br>European ancestry<br>(166,773 cases and 507,679 controls)	▪ https://ipsych.dk/en/research/downloads.<br>▪ doi: 10.1038/s41591-023-02352-1
Headache	N=224,073<br>European ancestry<br>(74,761 cases and 149,312 controls)	▪ https://figshare.com/articles/dataset/fourpainphenotypes1/7699556?file=14329373;<br>▪ doi: 10.1038/s41431-019-0530-2
Facial pain	N= 151,922<br>European ancestry<br>(2,610 cases and 149,312 controls)	▪ https://figshare.com/articles/dataset/fourpainphenotypes1/7699556?file=14329370;<br>▪ doi: 10.1038/s41431-019-0530-2
Neck/shoulder pain	N= 203,306<br>European ancestry<br>(53,994 cases and 149,312 controls)	▪ https://figshare.com/articles/dataset/fourpainphenotype2/7699583?file=14329418;<br>▪ doi: 10.1093/hmg/ddaa058
Back pain	N= 193,303<br>European ancestry<br>(4,3991 cases and 149,312 controls)	▪ https://figshare.com/articles/dataset/fourpainphenotypes1/7699556?file=14329367;<br>▪ doi: 10.1038/s41431-019-0530-2
Stomach pain	N= 157,529<br>European ancestry<br>(8,217 cases and 149,312 controls)	▪ https://figshare.com/articles/dataset/fourpainphenotypes1/7699556?file=14329364<br>▪ doi: 10.1038/s41431-019-0530-2
Hip pain	N= 159,428<br>European ancestry<br>(10,116 cases and 149,312 controls)	▪ https://figshare.com/articles/dataset/fourpainphenotype2/7699583?file=14329412;<br>▪ doi: 10.1038/s41431-019-0530-2
Knee pain	N= 171,516<br>European ancestry<br>(22,204 cases and 149,312 controls)	▪ https://figshare.com/articles/dataset/fourpainphenotype2/7699583?file=14329415;<br>▪ doi: 10.1038/s41431-019-0530-2
Pain all over the body (general pain)	N= 154982<br>European ancestry<br>(5670 cases and 149,312 controls)	▪ https://figshare.com/articles/dataset/fourpainphenotype2/7699583?file=14329421;<br>▪ doi: 10.1038/s41431-019-0530-2
Multisite chronic pain	N=387,649<br>European ancestry	▪ https://researchdata.gla.ac.uk/822/;<br>▪ doi: 10.1371/journal.pgen.1008164

*The GWAS was performed using data from Psychiatric Genomics Consortium (PGC). The original samples included N=480,395 participants of European ancestry (135,458 cases and 344,901 controls). We excluded the participants from UK Biobank (N=307,354) to minimize sample overlap. The samples from 23andMe (N=307,354) were also excluded due to general access constrains. The final samples included 45,591 cases and 97,674 controls.

** The GWAS was performed based on participants from iPSYCH2015, 23andMe, UK Biobank, FinnGen, and Million Veteran Program cohorts. The original samples included >1.3 million individuals of European ancestry. We excluded participants from UK biobank and 23andMe to avoid participant overlapping, leaving 166,773 cases and 507,679 controls.

Table S14. STROBE-MR checklist of recommended items to address in reports of Mendelian randomization studies

Item No.	Section	Checklist item	Page No.	Relevant text from manuscript
1	**TITLE and ABSTRACT**	Indicate Mendelian randomization (MR) as the study's design in the title and/or the abstract if that is a main purpose of the study	Abstract	Mendelian randomization supported potential causal inference.
	**INTRODUCTION**			
2	**Background**	Explain the scientific background and rationale for the reported study. What is the exposure? Is a potential causal relationship between exposure and outcome plausible? Justify why MR is a helpful method to address the study question	P2-3	As such, the reported associations between pain conditions and depression may have been affected by unmeasured confounders or reverse causation, necessitating the correct identification of causal relationships. MR uses genetic variants as instrumental variables and is less likely to be affected by residual confounding effects and reverse causality.
3	**Objectives**	State specific objectives clearly, including pre-specified causal hypotheses (if any). State that MR is a method that, under specific assumptions, intends to estimate causal effects	P3	Leveraging data from UK Biobank, we systematically investigated the prospective associations between pain conditions and depression risk and examined causal inferences using Mendelian randomization (MR). MR uses genetic variants as instrumental variables and is less likely to be affected by residual confounding effects and reverse causality.
	**METHODS**			
4	**Study design and data sources**	Present key elements of the study design early in the article. Consider including a table listing sources of data for all phases of the study. For each data source contributing to the analysis, describe the following:		

		a)	Setting: Describe the study design and the underlying population, if possible. Describe the setting, locations, and relevant dates, including periods of recruitment, exposure, follow-up, and data collection, when available.		Not applicable since this is a two-sample MR study based on summary-level data.
		b)	Participants: Give the eligibility criteria, and the sources and methods of selection of participants. Report the sample size, and whether any power or sample size calculations were carried out prior to the main analysis		Not applicable.
		c)	Describe measurement, quality control and selection of genetic variants		Not applicable.
		d)	For each exposure, outcome, and other relevant variables, describe methods of assessment and diagnostic criteria for diseases		Not applicable.
		e)	Provide details of ethics committee approval and participant informed consent, if relevant		The relevant ethical approval and participant consent has been obtained in original research.
5	**Assumptions**		Explicitly state the three core IV assumptions for the main analysis (relevance, independence and exclusion restriction) as well assumptions for any additional or sensitivity analysis	Supplement P27	See Table S7 legend.
6	**Statistical methods: main analysis**		Describe statistical methods and statistics used		
		a)	Describe how quantitative variables were handled in the analyses (i.e., scale, units, model)	P9	The number of co-occurring chronic pain sites was then calculated into a score ranging from 0 to 7.
		b)	Describe how genetic variants were handled in the analyses and, if applicable, how their weights were selected	P11	For each of the nine pain conditions (eight pain sites and MCP), we extracted significant single nucleotide polymorphisms (SNPs)

				based on the genome-wide significance threshold at P<5.0×10-8 and relaxed the threshold to P<5.0×10-7 for traits lacking enough SNPs (<3 SNPs).
		c) Describe the MR estimator (e.g. two-stage least squares, Wald ratio) and related statistics. Detail the included covariates and, in case of two-sample MR, whether the same covariate set was used for adjustment in the two samples		Used GWAS summary statistics.
		d) Explain how missing data were addressed		Not appliable.
		e) If applicable, indicate how multiple testing was addressed	P5	As we tested nine pain conditions, a two-sided P<0.05/9=0.0056 corrected by Bonferroni method was applied to indicate significant associations in the primary analysis.
7	**Assessment of assumptions**	Describe any methods or prior knowledge used to assess the assumptions or justify their validity	P12	We checked for evidence of heterogeneity and horizontal pleiotropy using Cochran's Q test and MR-Egger regression intercept term. We also applied MR Pleiotropy RESidual Sum and Outlier (MR-PRESSO) to detect and correct for any outliers.
8	**Sensitivity analyses and additional analyses**	Describe any sensitivity analyses or additional analyses performed (e.g. comparison of effect estimates from different approaches, independent replication, bias analytic techniques, validation of instruments, simulations)	P12	We also applied MR Pleiotropy RESidual Sum and Outlier (MR-PRESSO) to detect and correct for any outliers. Leave-one-SNP-out analysis was performed to assess if the overall effect was driven by any single SNP. For sensitivity analyses, we re-ran the MR analyses using summary statistics for depression from the Psychiatric Genomics Consortium of individuals of European descent, which did not include UK Biobank or 23andMe data.

9	**Software and pre-registration**				
		a)	Name statistical software and package(s), including version and settings used	P11	All analyses were conducted in R 4.3.3. We performed two-sample Mendelian randomization (MR) analyses to make causal inferences about the effects of genetically predicted pain conditions on depression using the "TwoSampleMR" package in R
		b)	State whether the study protocol and details were pre-registered (as well as when and where)		Not appliable.
	**RESULTS**				
10	**Descriptive data**				
		a)	Report the numbers of individuals at each stage of included studies and reasons for exclusion. Consider use of a flow diagram		Not appliable.
		b)	Report summary statistics for phenotypic exposure(s), outcome(s), and other relevant variables (e.g. means, SDs, proportions)	Supplement<br>P27	See Table S7.
		c)	If the data sources include meta-analyses of previous studies, provide the assessments of heterogeneity across these studies		Not appliable.
		d)	For two-sample MR:<br>i. Provide justification of the similarity of the genetic variant-exposure associations between the exposure and outcome samples<br>ii. Provide information on the number of individuals who overlap between the exposure and outcome studies	P11	Not appliable.<br>No overlap.

11	**Main results**			
		a) Report the associations between genetic variant and exposure, and between genetic variant and outcome, preferably on an interpretable scale		Not appliable.
		b) Report MR estimates of the relationship between exposure and outcome, and the measures of uncertainty from the MR analysis, on an interpretable scale, such as odds ratio or relative risk per SD difference	Supplement P25-26	See Table S6.
		c) If relevant, consider translating estimates of relative risk into absolute risk for a meaningful time period		Not appliable.
		d) Consider plots to visualize results (e.g. forest plot, scatterplot of associations between genetic variants and outcome versus between genetic variants and exposure)	Supplement P10-11	See Figure 3D, Fig. S9, Fig. S10.
12	**Assessment of assumptions**			
		a) Report the assessment of the validity of the assumptions	P5	The MR Egger intercept test suggested no obvious directional pleiotropy (intercept=0.012, P= 0.056), but Cochran's Q test indicated significant heterogeneity (Q=106.02, P=5.95×10-12). Therefore, the IVW method under random effect was used as the primary method.
		b) Report any additional statistics (e.g., assessments of heterogeneity across genetic variants, such as I^2, Q statistic or E-value)	P5	The MR Egger intercept test suggested no obvious directional pleiotropy (intercept=0.012, P= 0.056), but Cochran's Q test indicated significant heterogeneity (Q=106.02, P=5.95×10-12). Therefore, the

					IVW method under random effect was used as the primary method.
13	**Sensitivity analyses and additional analyses**				
		a)	Report any sensitivity analyses to assess the robustness of the main results to violations of the assumptions	P5	Moreover, MR-PRESSO detected four outlying variants and removing these outliers nominally impacted the estimation (OR=2.06, 95% CI= [1.70, 2.50], P=2.20×10-13).
		b)	Report results from other sensitivity analyses or additional analyses	P5	Analyses leaving out each SNP revealed that no single SNP drove the estimation (Figure S7). These results were consistent with those using summary statistics for depression from a different consortium (Figure S8, Table S10).
		c)	Report any assessment of direction of causal relationship (e.g., bidirectional MR)		Not appliable.
		d)	When relevant, report and compare with estimates from non-MR analyses		Not appliable.
		e)	Consider additional plots to visualize results (e.g., leave-one-out analyses)	Supplement<br>P10	See Fig. S9.
	**DISCUSSION**				
14	**Key results**		Summarize key results with reference to study objectives	P7	Furthermore, using MR with genetic instruments selected from large-scale GWAS, we found evidence supporting a potential causal effect for genetically predicted MCP, but not for any site-specific pain, on depression.
15	**Limitations**		Discuss limitations of the study, taking into account the validity of the IV assumptions, other sources of	P8	Second, although the largest existing GWAS of pain conditions were used, some identified

		potential bias, and imprecision. Discuss both direction and magnitude of any potential bias and any efforts to address them		few significant SNPs and did not explain a significant amount of the total variance. These SNPs may not be exact proxies of pain conditions. Thus, updated MR analyses are needed as more powered genetic discoveries emerge.
16	**Interpretation**			
	a)	Meaning: Give a cautious overall interpretation of results in the context of their limitations and in comparison with other studies	P7	Our causal estimation was like that observed in two recent MR studies. Nevertheless, overfitting might be a common concern in these studies because they used overlapped samples to generate summary statistics for pain and depression.
	b)	Mechanism: Discuss underlying biological mechanisms that could drive a potential causal relationship between the investigated exposure and the outcome, and whether the gene-environment equivalence assumption is reasonable. Use causal language carefully, clarifying that IV estimates may provide causal effects only under certain assumptions	P8	This result highlights the importance of a comprehensive approach to chronic pain patients accounting for all painful body sites and suggests that the development of depression may stem from the cumulative effect of multiple overlapping pain conditions.<br><br>A direct study of the causal relationship between MCP and depression requires further confirmation before it is targeted in resource-intensive trails.
	c)	Clinical relevance: Discuss whether the results have clinical or public policy relevance, and to what extent they inform effect sizes of possible interventions	P8	Our findings have potential implications for public health and clinical care. Because of the significant increase in the risk of depression in individuals with pain, early screening for pain conditions may be an effective way to identify high-risk individuals who may benefit from intensive preventive efforts.

17	**Generalizability**	Discuss the generalizability of the study results (a) to other populations, (b) across other exposure periods/timings, and (c) across other levels of exposure	P9	Fifth, data in UK Biobank are limited to people of European white ancestry aged 40 years and over. Considering evidence suggesting ethnic differences in pain pathophysiology, replication and generalization of the observed associations are essential when more diverse samples become available.
	**OTHER INFORMATION**			
18	**Funding**	Describe sources of funding and the role of funders in the present study and, if applicable, sources of funding for the databases and original study or studies on which the present study is based	P18	Presented in Funding section.
19	**Data and data sharing**	Provide the data used to perform all analyses or report where and how the data can be accessed, and reference these sources in the article. Provide the statistical code needed to reproduce the results in the article, or report whether the code is publicly accessible and if so, where	P19	Presented in Data and materials availability section.
20	**Conflicts of Interest**	All authors should declare all potential conflicts of interest	P19	The authors declare no competing interests.
